# The hemodynamic complexities underlying transient ischemic attacks in early-stage Moyamoya disease: an exploratory CFD study

**DOI:** 10.1038/s41598-020-60683-2

**Published:** 2020-02-28

**Authors:** Sherif Rashad, Khalid M. Saqr, Miki Fujimura, Kuniyasu Niizuma, Teiji Tominaga

**Affiliations:** 1 Department of Neurosurgical Engineering and Translational Neuroscience, Tohoku University Graduate School of Medicine, Sendai, Miyagi 980-8575 Japan; 2 Department of Neurosurgery, Tohoku University Graduate School of Medicine, Sendai, Miyagi 980-8575 Japan; 3 Mechanical Engineering Department, College of Engineering and Technology, Arab Academy for Science, Technology and Maritime Transport, 1029 Abu-Kir Alexandria, Egypt; 4 Department of Neurosurgery, Kohnan hospital, Sendai, Miyagi Japan; 5 Department of Neurosurgical Engineering and Translational Neuroscience, Graduate School of Biomedical Engineering, Tohoku University, Sendai, Japan

**Keywords:** Stroke, Fluid dynamics

## Abstract

Moyamoya disease (MMD) is a rare cerebro-occlusive disease with unknown etiology that can cause both ischemic and hemorrhagic stroke. MMD is characterized by progressive stenosis of the terminal internal carotid artery (ICA) and development of basal brain collaterals. Early-stage MMD is known to cause hemodynamic insufficiency despite mild or moderate stenosis of the intracranial arteries, but the exact mechanism underlying this pathophysiological condition is undetermined. We used high-resolution Large Eddy Simulations to investigate multiple complex hemodynamic phenomena that led to cerebral ischemia in five patients with early-stage MMD. The effects of transitional flow, coherent flow structures and blood shear-thinning properties through regions of tortuous and stenosed arteries were explored and linked to symptomatology. It is evidently shown that in some cases complex vortex structures, such as Rankine-type vortices, redirects blood flow away from some arteries causing significant reduction in blood flow. Moreover, partial blood hammer (PBH) phenomenon was detected in some cases and led to significant hemodynamic insufficiency. PBH events were attributed to the interaction between shear-thinning properties, transitional flow structures and loss of upstream pressure-velocity phase lag. We clearly show that the hemodynamic complexities in early-stage MMD could induce ischemia and explain the non-responsiveness to antiplatelet therapy.

## Introduction

Moyamoya disease (MMD) is a rare cerebrovascular disease, characterized by bilateral occlusive changes at the internal carotid artery (ICA) terminus and an abnormal collaterals development at the base of the brain^1^. MMD is more prevalent in East Asian populations and can present as either ischemic or hemorrhagic stroke^2^. MMD can occur in pediatric or adult populations^2–4^ and can present as bilateral or unilateral disease involvement^5^. The basic pathology of MMD, including its temporal profile, is clearly indicated by Suzuki’s angiographic staging^1^, demonstrating the pathologic conversion of the cerebral vascular supply from internal carotid (IC) to the external carotid (EC) system^6^. Insufficiency of this ‘*IC-EC conversion system*’ could result in either ischemic or hemorrhage MMD presentations.

Recent advance in high resolution magnetic resonance (HR-MR) vascular wall imaging enabled us to diagnose MMD even in its early stage, by demonstrating the characteristic vascular wall structure such as outer-diameter narrowing and the concentric stenosis of the affected intracranial arteries^7–9^. All of these characteristic findings by HR-MR vascular wall imaging are precisely reflected by vascular wall pathology of MMD including medial layer thinning and the waving of internal elastic lamina^10,11^. Accurate diagnosis of the early-stage MMD is clinically important because it could manifest as transient ischemic attacks (TIA) and/or cerebral infarction due to the significant hemodynamic compromise, despite its mild or moderate stenosis. Nonetheless, the mechanism underlying the hemodynamic insufficiency in early-stage MMD is totally undetermined. To address this critical issue, we investigated the hemodynamic complexities of early-stage MMD by analyzing five MMD patients presenting with progressive ischemia despite their mild or moderate stenosis of the affected arteries using high-fidelity computational fluid dynamics (CFD) simulations. We hypothesized that studying hemodynamic phenomena could provide valuable information to delineate the underlying pathology and symptomatology of early-stage MMD since CFD has been a valuable tool that provided critical information to evaluate the relationship between the pathobiology of the vascular wall anatomy and the blood hemodynamics in variety of cerebrovascular diseases^12^.

Several vascular diseases had their hemodynamic features studied using computational fluid dynamics (CFD) to understand the relationship between the pathobiology of endothelial cells and the vessel wall and blood hemodynamics and physics^12^. There is a wealth of publications for example on cerebral aneurysms, carotid stenosis and intracranial stenosis^12–17^. However, a Scopus search using the terms; “Moyamoya” + “computational fluid dynamics” or “CFD” reveals less than 10 publications that explored the blood dynamics features associated with MMD but with no possible link to early MMD pathology or symptomatology.

In this work we show, for the first time, the hemodynamic complexities in early-stage MMD by analyzing 5 cases presenting with progressive ischemia using high-fidelity CFD simulations. We show a mechanistic relationship between the results of CFD simulations and ischemia development in these patients. Precisely, we show the existence of a partial blood hammer (PBH) phenomenon resulting from the complex fluid dynamics of pulsatile non-Newtonian flow in tortuous and stenosed arterial geometries that can explain the non-responsiveness of their symptoms to antiplatelet medical therapy. Moreover, we show for the first-time dynamic changes in viscosity induced by the complex arterial geometry that lead to spatiotemporally persistent flow stagnation and recirculation zones with shear-rate conditions that can possibly induce platelet aggregation and subsequent embolism. Finally, we report for the first time the presence of Rankine vortex^18–20^ (a type of forced vortex similar to tornado core) in the cerebral circulation and its effect on blood flow and ischemia development.

## Results

### Clinical data and patient outcome

Initially 11 patients with early-stage MMD (Suzuki grade I and II^1^) that had presented with TIA or ischemic stroke were retrospectively found in our database. However, only 5 patients had undergone 3D angiography and thus STL models could be created for these patients with adequate quality for high-fidelity CFD analysis. Three patients presented with TIA and two patients presented by ischemic stroke. All 5 patients had atypical early-stage MMD and were diagnosed initially as having suspicious MMD based on pattern of vessel involvement and reduced cerebral perfusion observed with MRI. Three patients had bilateral disease and two patients had unilateral disease involvement. Patients were operated upon by extracranial-intracranial bypass (superficial temporal artery (STA)-middle cerebral artery (MCA) bypass)^3,4,21,22^. While the presence of the characteristic Moyamoya collaterals was not very evident in these cases, the surgery was justified based on the JET criteria for extracranial-intracranial bypass surgery^23^ and based on cerebral perfusion insufficiency rather than a definitive MMD diagnosis. Intraoperatively, the cerebral arteries were observed to have thin vascular wall and no atherosclerotic lesions were observed, further confirming the diagnosis of MMD. Postoperatively all patients recovered and had no further symptoms and cerebral blood flow (CBF) was improved 1 and 7 days on MR-SPECT (data not shown). One year follow up showed improvement in cerebral blood flow. Interestingly, one year follow up MR angiography revealed a total occlusion of the terminal ICA in most of the patients, which may be a part of the normal pathophysiologic progression course of MMD^4^. Patients’ clinical data, clinical imaging and STL models’ images are presented in Supplementary Table 3 and Supplementary Figs. 2 to 11

### Complex hemodynamic phenomena observed in MMD patients

**Figure 1:**
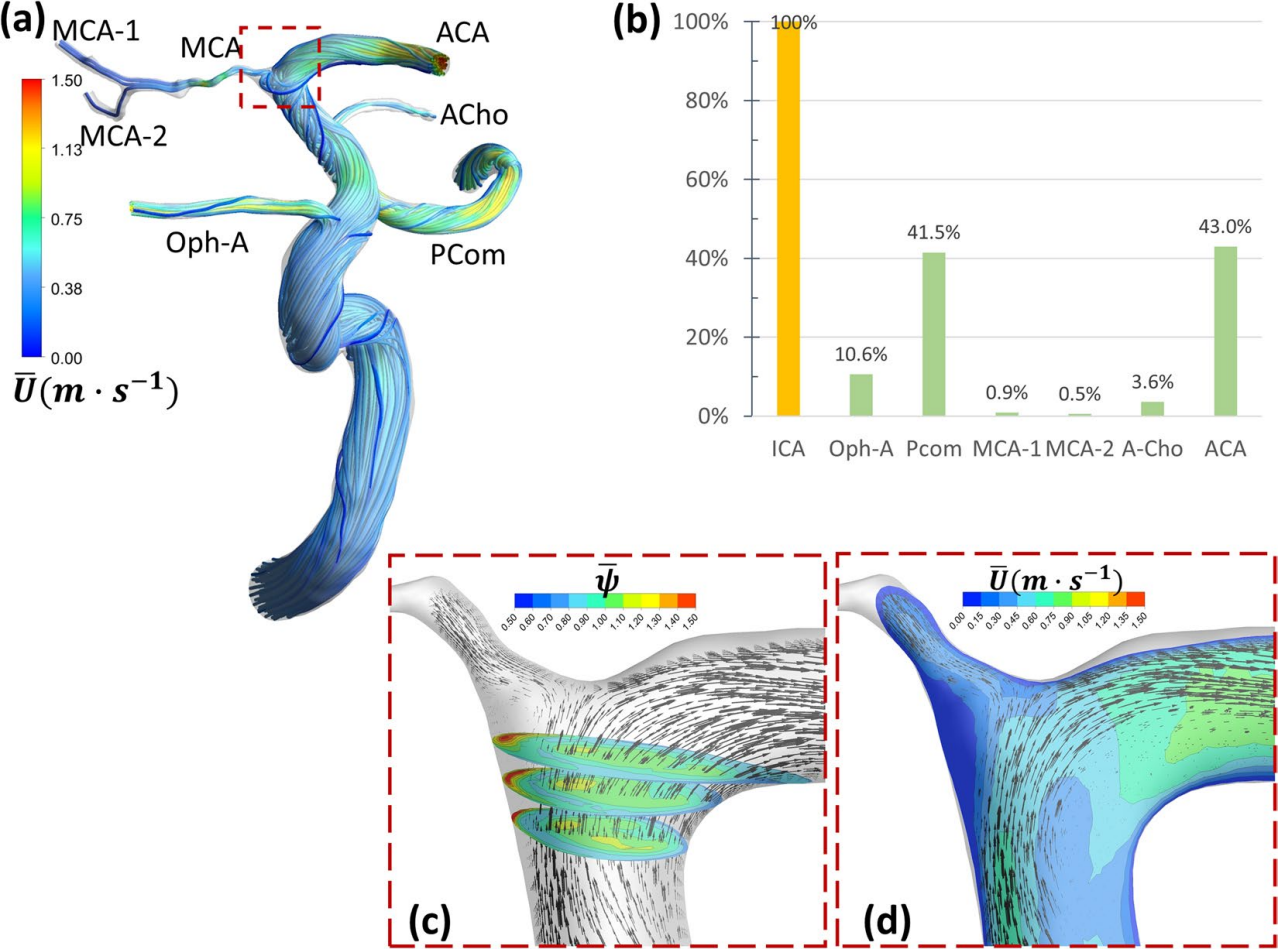
Case 1: MCA bifurcation undergoes flow choking and complex vortex structure. (**a**) Time-averaged velocity path-lines showing the development of complex swirl and vortex structures in the ICA and downstream at the ICA bifurcation. (**b**) Percentage of time-averaged flow rate reduction in the MCA bifurcation which shows choked flow at MCA outlets; MCA-1 and MCA-2. (**c**) 2D contours of $\bar{\psi }$ showing increased viscosity near to the entry of the MCA. (**d**) Time averaged velocity field depicted on the mid-plane showing stagnation in the high-viscosity region causing separation until the bifurcation apex. Note the reduced number and size of arrows representing the flow at the area of flow stagnation and increased viscosity in c and d. ICA: internal carotid artery, MCA: middle cerebral artery, ACA, anterior cerebral artery, Oph-A: Ophthalmic artery, Pcom: posterior communicating artery, MCA-1 and MCA-2: M2 branches of the MCA.

**Figure 2:**
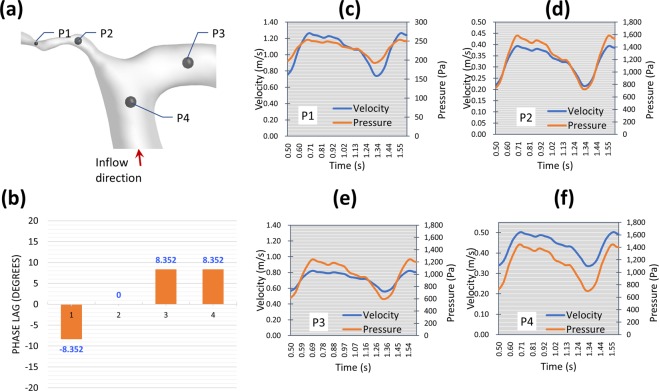
Case 1: Comparison of the phase lag between velocity and pressure waveforms explains the characteristics of the choked artery and PBH. (**a**) Location of four points were selected to investigate the phase lag. (**b**) Phase lag in degrees as measured from the LES results showing zero-phase lag at the MCA associated with sharp change in geometry. This zero phase-lag point is the point at which PBH, and flow chocking take place. (**c–f**) Depiction of the velocity and pressure history during one pulse showing the variation in phase lag along geometry.

**Figure 3:**
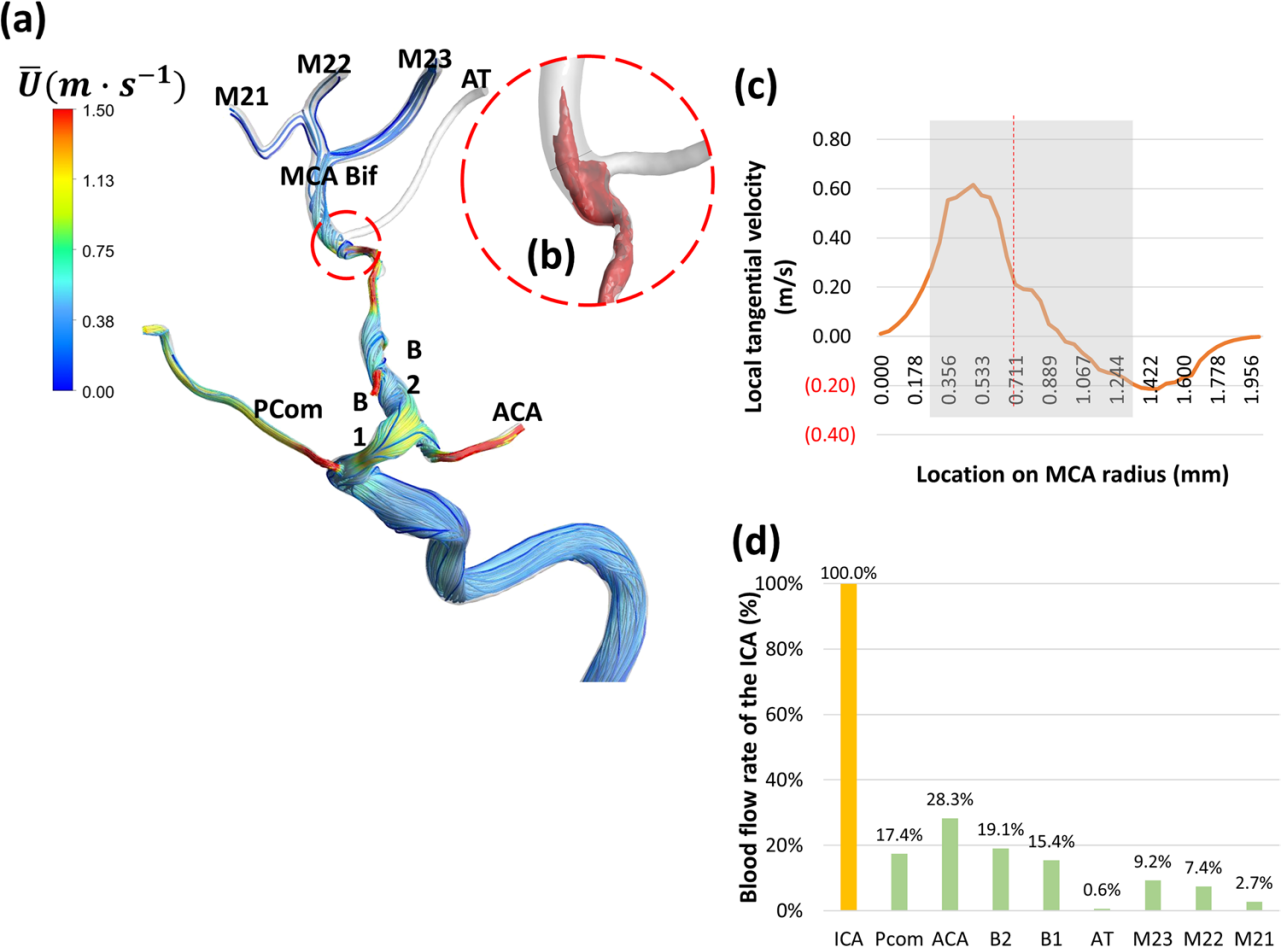
Case 2: Rankine vortex prevents blood flow from feeding the AT and choking occurs at the MCA bifurcation (**a**) Time-averaged flow path-lines colored by time-averaged mean velocity. Note the increased velocity in the stenosed MCA segment. (**b**) Iso-volume of Q-criterion at threshold value (Q = 0.05) at the origin of the AT artery showing the core of a Rankine-type vortex. (**c**) Profile of time-averaged local tangential velocity at the post-stenotic segment of the MCA showing a typical structure of asymmetric Rankine vortex with a forced vortex core (depicted by the grey shading) and free vortex outer rim with negative velocity marking a precessing recirculation zone. The red-colored values on the Y-axis represent velocity in the opposite direction. (**d**) Percentage blood flow rate of the ICA and its branches showing reduced blood flow at the anterior temporal (AT) artery. B1: a small branch from MCA. B2: a small branch from MCA. M21, M22 and M23: Outflows from the M2 segment of the MCA.

**Figure 4:**
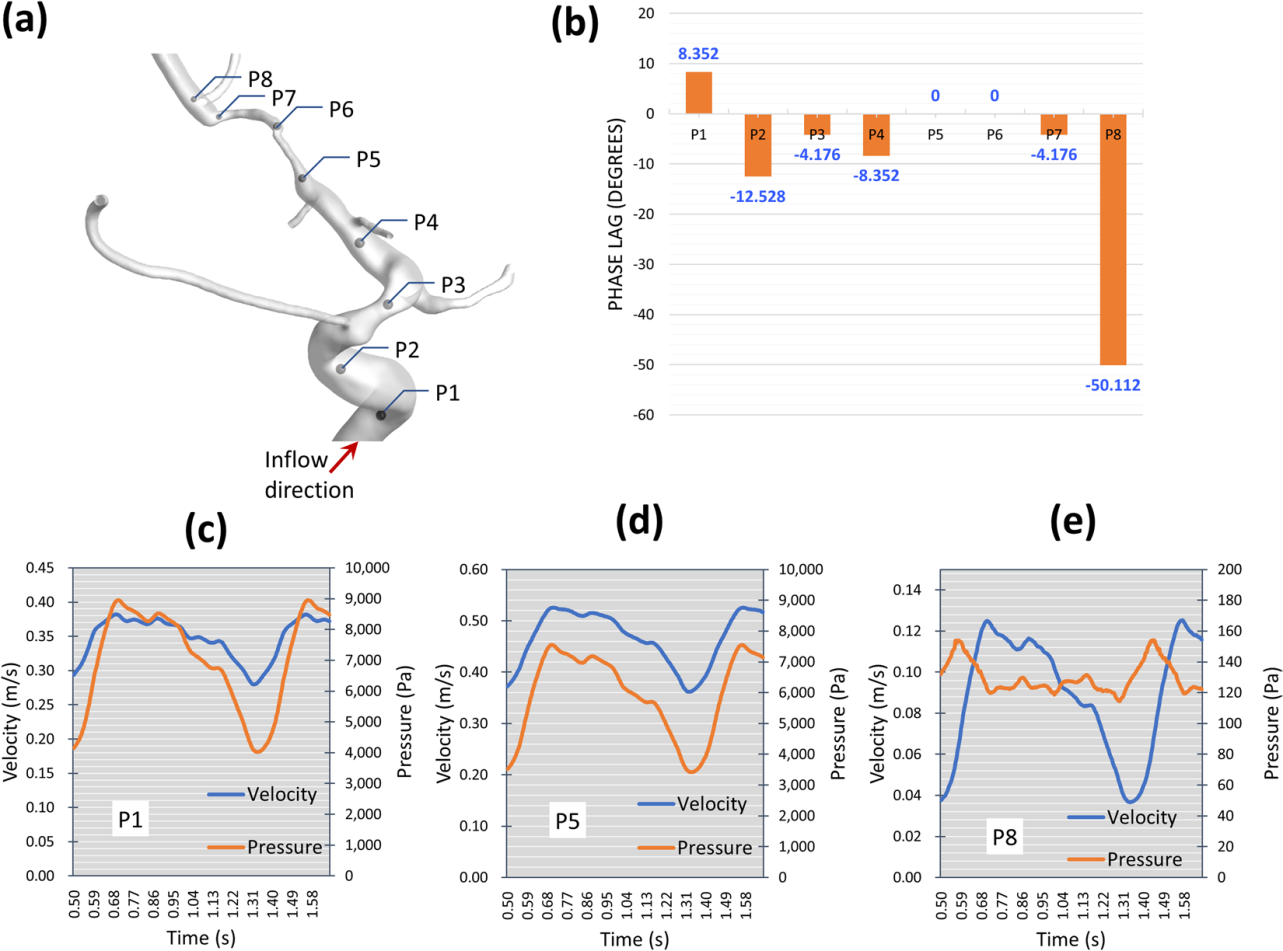
Case 2: Observation of the phase lag between pressure and flow waveforms explains the location of flow choking in the MCA. (**a**) Eight locations taken along the centerline of the ICA. (**b**) comparison of the phase lag at the eight locations in degrees shows zero phase lag at locations P5 and P6. (**c**–**e**) detailed comparison of the phase lag at three representative locations. The left and right y-axis represent velocity and pressure scales in m/s and pa, respictively (note the difference in velocity scale between (**c**,**d**).

**Figure 5:**
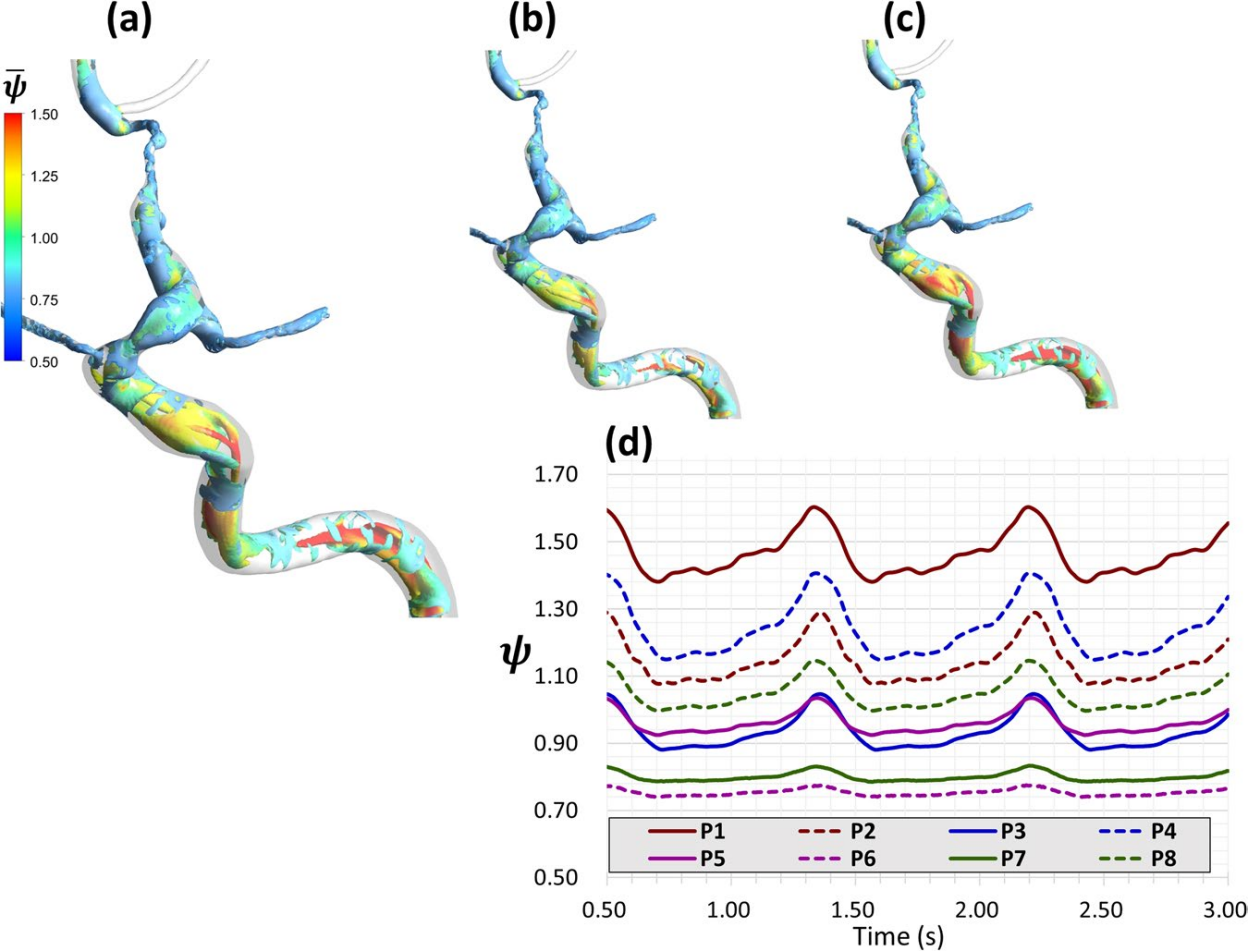
Case 2: Complex vortex structure persisting in the flow shown by depicting the time-averaged and instantinous Q-criterion and non-Newtonian viscosity history. (**a**) Iso-volumes (Q = 0.01) of time averaged Q-criterion, coloured by $\bar{\psi }$. (**b**,**c**) Iso-volumes of instantinous Q-criterion in the peak systole and end diastole, respictively, following the same colour map of (**a**). (**d**) History of instantinous $\psi$ in the eight locations depicted in Fig. (4a) for 2.5 seconds showing large variation between the flow choking locations (P5, P6) and the upstream flow in the ICA in terms of instantaneous viscosity.

**Figure 6:**
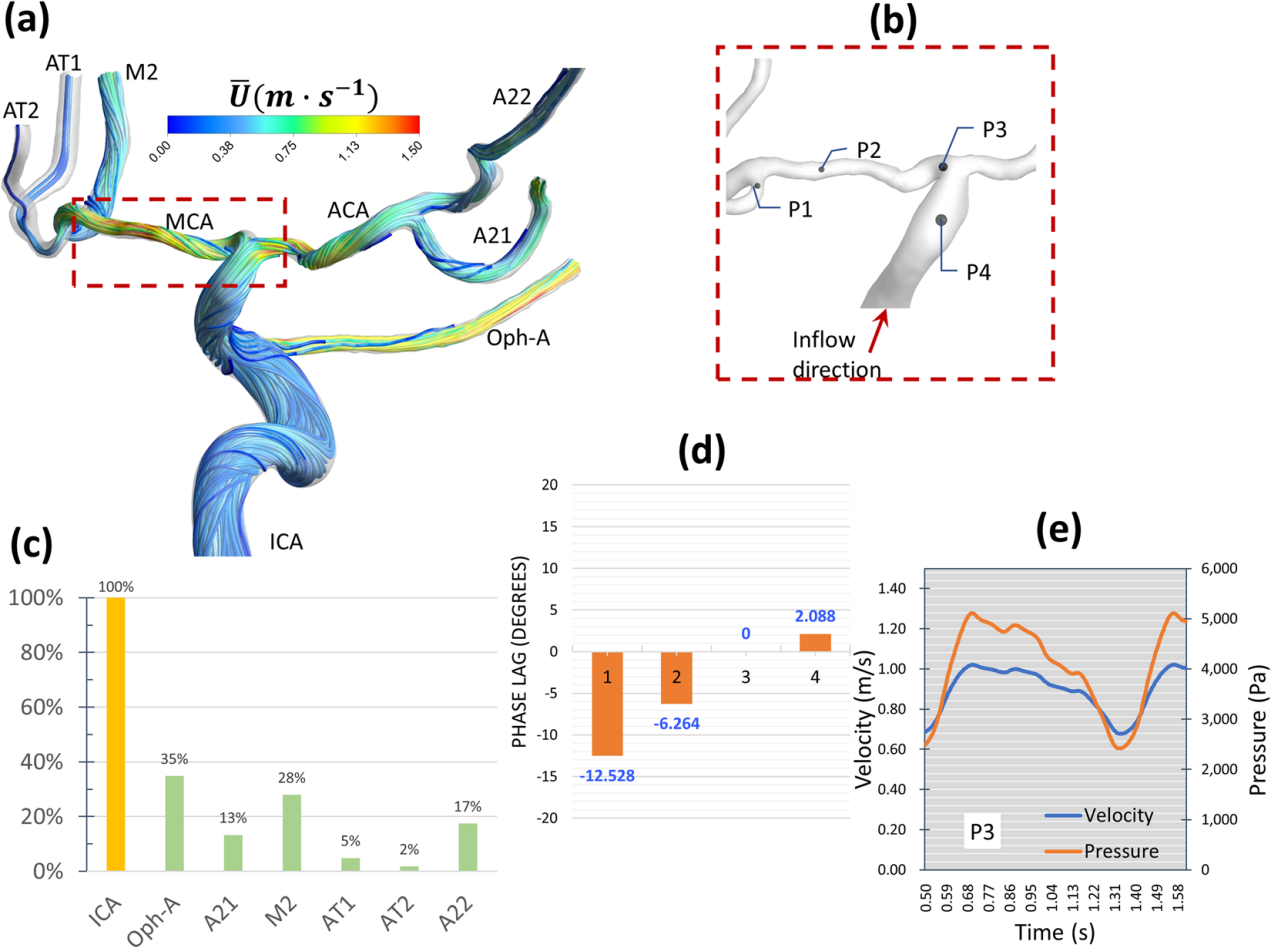
Case 3: Swirl development along the ICA and through the stenosed tortous entry of the MCA leads to flow choking. (**a**) Time-averaged flow pathlines showing the complexity of swirl and vortex structure developeng along the ICA and branches. (**b**) Four locations selected on the center of the ICA, MCA and at the inlet of AT2 (note the change in view angle). (**c**) Percentage loss of flow rate at the AT2 and AT2 due to flow choking compared with other branches. (**d**) Zero phase lag at the stenosed tortous entry of the MCA (P3) in comparison with leading and lagging pressure waves at other locations. (**e**) Velocity and pressure profiles at P3 shows the loss of phase lag to zero value. AT1 and AT2: anterior temporal artery branches.

**Figure 7:**
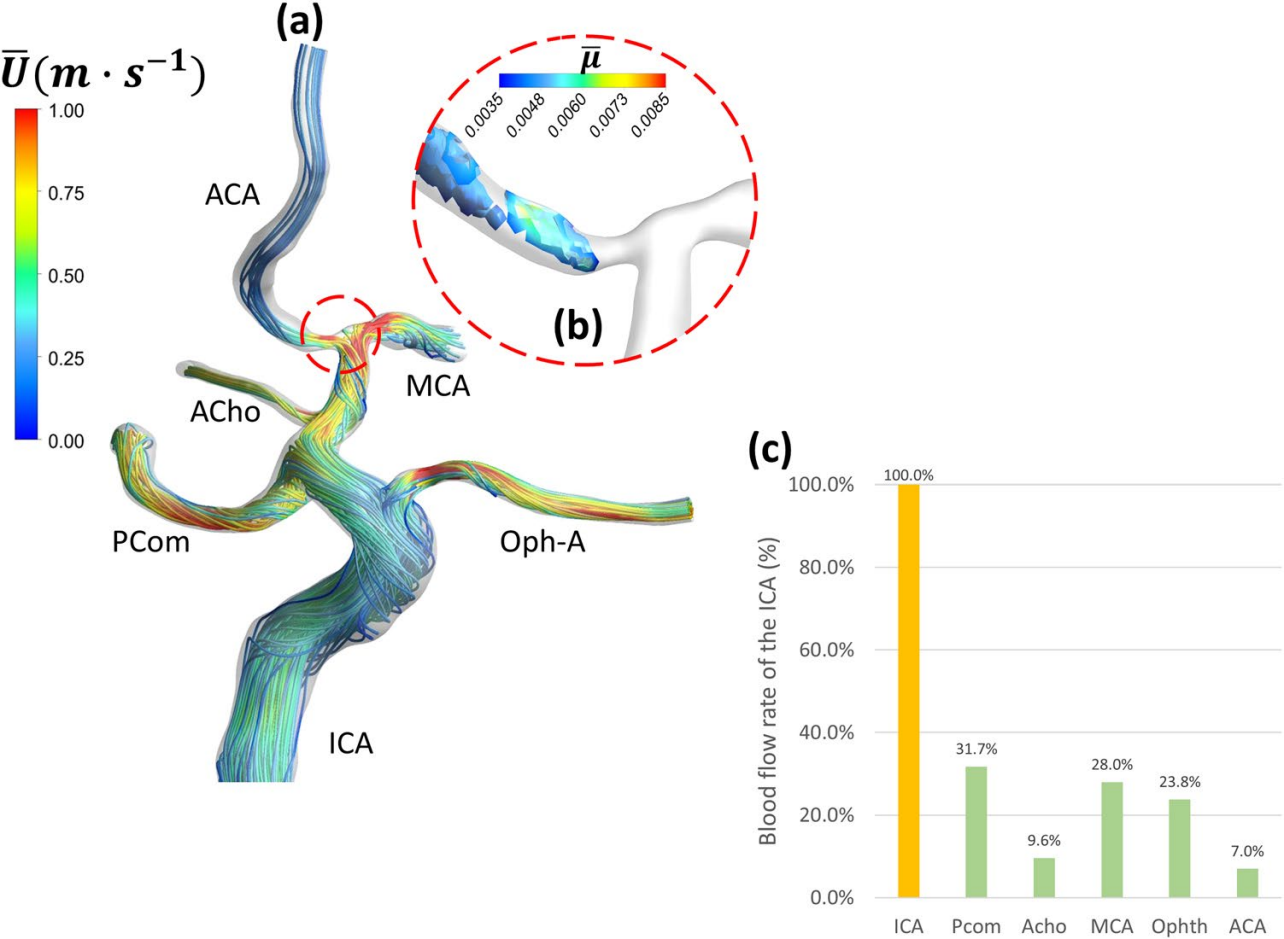
Case 4: Persisting region of hemodynamically induced high viscosity at the entry of ACA causes reduction in time-averaged blood flow rate (**a**) time-averaged flow pathlines showing low velocity in the ACA. (**b**) Iso-volumes of time-averaged non-Newtonian viscosity showing 50~70% increase in the viscosity in persisting structure at the entry of ACA, possibly induced by post-stenotic swirl flow. (**c**) Comparison of time-averaged blood flow rate through different branches.

The hemodynamic phenomena observed in MMD patients were not uniform in all patients. Some patients expressed all the phenomena observed in our analysis and thus their ischemic symptoms can be a sum of all the phenomena or a result of one dominating phenomena, while other patients had only one evident phenomenon that can explain their symptoms. In this section we describe the main observations and discuss their relevance and details in the discussion section. [Case 1: Figs. 1 and 2 and Supplementary Fig. 12. Case 2: Figs. 3–5. Case 3: Fig. 6. Case 4: Fig. 7. Case 5: Supplementary Fig. 13].

#### Time-averaged flow structure

The first step in exploring the hemodynamic complexities of MMD is to observe the flow structure in such complex tortuous arterial morphologies. The flow field variables vary significantly through each cardiac pulse; however, the time-averaged values present general description of the main flow features. Three-dimensional pathlines of time averaged velocity $(\bar{U}=\frac{1}{T}{\int }_{t}^{T}\tilde{u}({x}_{i},t)dt)$ were plotted to depict a general description of the flow. As shown in Figs. 1a, 3a, 6a, 7a and Supplementary Fig. 13a; the flow is dominated by tortuosity induced swirl with highly curved streamlines. In some parts of the highly tortuous and stenosed segments (Figs. 3a, 7a) the time-averaged velocity reaches maximum values. Separation and stagnation regions are detected in Fig. 1c,d, respectively, showing the effect of MMD morphology on flow structure.

#### Tortuosity induced swirl and rankine vortex phenomena

In the current work, Rankine vortex was found in some arterial segments in all cases. However, it was found to cause passive flow control leading to blood flow insufficiency only in two cases; namely 2 and 3. In case 2 (shown in Fig. 3), a Rankine vortex is induced by complex morphology at the origin of the anterior temporal (AT) artery. The complex morphology is characterized by tortuous sudden expansion region which induced the vortex core. Fig. 3b shows an isovolume of the vortex core denoted by positive Q-criterion while Fig. 3c shows the tangential velocity profile along the radius of this segment. The flow, therefore, rotates tangential to the inlet plane of the AT artery. The effect of Rankine vortex in this case is clear by depicting the insufficient time-averaged blood flow percentage of AT, as shown in Fig. 3d, where AT only receives 0.6% of the ICA flow rate.

#### Observation of Partial blood hammer (PBH) phenomenon

*Blood hammer*^24^ is a borrowed term from the hydraulics field. It describes an analogous phenomenon to the well-known *water hammer*^25^ phenomenon in fluid pipelines. The definition of blood hammer is a sudden and transient increase in upstream pressure due to corresponding transient abrupt increase in downstream pressure. It has been previously reported in some cases of cerebral hemodynamics^26^. However, it is noteworthy to state that the analytical and exact models of the *hammer* phenomena reported in literature do not include the effects of streamline curvature nor non-Newtonian viscosity^27^. In this work, partial blood hammer (PBH) was observed as a loss of phase-lag between flow and pressure waveforms in cases 1, 2, and 3. In the present work it occurs due to the change in hydrodynamic resistance resulting from the interaction between non-Newtonian viscosity and turbulence fields past a complex stenosed arterial segment. In Fig. 2 it was observed shortly after the inlet plane of the MCA where the flow is restricted in a sharp stenosed curved segment. While in Fig. 4, it was observed at two points within the stenosed ICA segment (Fig. 4b,c), where the flow is passing a highly tortuous and stenosed segment. In Fig. 6, PBH is observed at inlet plane of MCA, where the flow is dominated by swirl and passing through a stenosed region. In all cases, the loss of phase-lag marks maximum hydraulic resistance downstream the PBH location.

#### Interaction between coherent turbulent structures and non-Newtonian viscosity

Coherent structures are marked by low-frequency rotational and dissipative motion. In the present work, coherent structures were detected by the Q-criterion which is the second invariant of the velocity gradient tensor^28^. In non-Newtonian flow, coherent structures interact with the viscosity with complex dynamics that is still under investigation^29^. Fig. 1d shows a region of high viscosity $(\psi \approx 1.5)$ associated with the separation zone upstream the stenosed MCA. This region is persistent in time and stationary in space. Fig. 5a–c shows isovolumes of positive Q-criterion levels colored by $\psi$ to highlight the latter interaction. Various structures such as Dean^30,31^ and hairpin vortices can be observed with varying $\psi$ values. The time variation of viscosity along the ICA upstream and through the stenosed region, denoted by $\psi$, is shown in Fig. (5d). It can be seen that the viscosity varies from −30% to +60% with respect to the Newtonian viscosity value. Figure 7d shows persistent and stationary regions of elevated viscosity at the inlet segment of the anterior cerebral artery (ACA), which results in moderate reduction of ACA flow rate, as shown in Fig. (7c). In Supplementary Fig. 13, case 5 shows persisting stagnation regions directly downstream the inlet planes of the MCA and ACA. These regions are deemed to exist because of the shear-thinning blood properties which increases the viscosity creating local high viscous resistance to the flow.

#### Case specific hemodynamic phenomena

While the above mentioned flow phenomena were observed more or less in all patients, their final contribution to the patients’ symptomatic presentation was not homogenous amongst all patients, with some patients experiencing hemodynamic insufficiency from only one phenomena and others experiencing a collection of two or three phenomena at a critical point in the ICA bifurcation causing hemodynamic insufficiency and TIAs. Here we review each case and highlight the possible contributing mechanisms leading to ischemia development.

**Case 1 (**Figs. 1 and 2 and Supplementary Figs. 2, 3 and 12**):** This patient presented with a moderate sized cerebral infarction resulting from cerebral hemodynamic insufficiency to the right hemisphere (Supplementary Fig. 2). He was finally diagnosed as having a unilateral MMD of grade II on the right side. Blood flow insufficiency was detected by CFD in M2 branches (dubbed MCA-1 and MCA-2 in Fig. 1) (Fig. 1b). The inflow to MCA was reduced due to a coherent region of high viscosity upstream to the MCA inlet plane (Fig. 1c,d). Then, PBH was detected in the stenosed segment of the MCA, which caused pressure to rise at the first segment of the MCA reducing time-averaged blood flow rate (Fig. 2). The combination of PBH and increased viscosity led to severe reduction of flow rate through the MCA (Fig. 1b) which can explain the ischemic symptoms and also the non-responsiveness to best medical therapy (BMT).

**Case 2 (**Figs. 3–5, Supplementary Figs. 4 and 5): This patient presented by progressive TIAs not responding to BMT. MRI SPECT showed a right hemispheric hemodynamic insufficiency in the MCA territory at rest (Supplementary Fig. 4c). The patient was diagnosed as right grade II MMD, based on the symptoms and the presence of definitive MMD of grade III on the left side. Drastic blood flow insufficiency was detected at the AT artery (0.6% of the ICA blood flow) (Fig. 3d). A Rankine vortex was detected in the vicinity of the AT inlet plane. The Rankine vortex prevented mass transfer normal to its axis, therefore, blood was not allowed to the AT (Fig. 3b,c). In other words, the Rankine vortex only allows blood flow along its axis, and since the AT origin was perpendicular to its long axis, blood could not flow into the AT. In addition, PBH was detected in two points in the stenosed MCA segment resulting in increased flow resistance upstream the MCA (Fig. 4). It was also found that coherent turbulent structures interact dynamically with non-Newtonian viscosity leading to areas of alternating high and low viscosity in the ICA and MCA (Fig. 5). Indeed, these areas showed variations between peak systole (Fig. 5b) and end diastole (Fig. 5c) as showed when plotted over time in Fig. 5d. While the dynamic viscosity did not seem to interfere with the blood flow across the MCA in this case, the existence of PBH and Rankine vortex together led to complex hemodynamic based flow insufficiency that would not have been corrected by BMT and would explain the progression of symptoms.

**Case 3 (**Fig. 6 and Supplementary Figs. 6 and 7): This patient presented with ischemic infarction despite being diagnosed with grade I MMD on the right side. Blood flow insufficiency was detected in AT artery (dubbed as AT1 and AT2 in Fig. 6) (Fig. 6b). PBH was detected upstream the MCA, in a region where MCA exhibits stenosis and complex tortuosity and curvature (Fig. 6d,e). In addition, a Rankine vortex was detected at the bifurcation plane between MCA and AT (Data not shown). The existence of both phenomena can very well explain the extreme hemodynamic insufficiency despite an early disease of only grade I. Moreover, both hemodynamic phenomena can also explain the non-responsiveness to medical therapy.

**Case 4 (**Fig. 7 and Supplementary Figs. 8 and 9): This patient presented with a left recurrent TIAs and was diagnosed as having a bilateral MMD of grade II on both sides. The ACA outflow was found to be only 7% of the ICA blood flow. By exploring the persisting viscous structures, it was found that there is a region of high viscosity at the inlet of the ACA, probably due to the coherent turbulence structures, and resulting in increasing the flow resistance upstream of the ACA. This increased turbulent viscosity would indeed impede the cerebral blood flow ultimately resulting in TIAs.

**Case 5** (Supplementary Figs. 10, 11 and 13): This case also presented with TIAs and was diagnosed as having unilateral MMD of grade II on the left side. The complex morphology of the stenosed MCA-AT bifurcation results in creating separation and stagnation zones at the inlet of both arteries. In addition, the severe curvature of the ACA results in creating stagnation and high-viscosity region, subsequently increasing the flow resistance and reducing ACA flow to 10% of the ICA flow.

## Discussion

Since the description of Moyamoya disease in the 1960s by Professor Jiro Suzuki^1^, it has gained a lot of attention. However, the hemodynamic changes occurring during early stages of MMD are still unveiled. We show in this work, by using high fidelity CFD simulations, that several hemodynamic phenomena can occur in early-stage MMD. These phenomena can explain the symptomatology of early-stage MMD induced progressive stroke, that is resistant to best medical therapy and cannot be explained solely on the basis of the degree of arterial stenosis.

The first phenomena that explains blood flow insufficiency in our work is the PBH. Blood hammer was actually studied in theory in the cerebral circulation as early as the 1970s^32^, however it remains an understudied phenomena that has not been fully characterized in patient specific geometries. In the vast majority of previous studies, total *blood hammer* was only studied in steady Newtonian flow subjected to transient change in hydrodynamic resistance. Very few studies discussed PBH in different settings involving elastic boundaries^33^ or transient flow blockage^34,35^. In the present work, the nonlinearity causing transient changes in the hydrodynamic resistance is caused by the non-Newtonian viscosity^36^. The presence of PBH is very clear in the pressure coefficient comparison presented in the example in Supplementary Fig. 12. It must be noted, however, that the physics of transitional/turbulent non-Newtonian flow is not fully comprehended until the present time. It must be also noted that transient phenomena such as PBH in non-Newtonian flows have not been sufficiently investigated in theoretical fluid dynamics literature. Theoretically, if a give patient suffers from repeated TIAs due to PBH, antiplatelet therapy will not improve the condition, due to the physical nature of the phenomena and its independence from platelet aggregation. This clearly shows the value of assessing this phenomenon, not only in MMD, but also in other cause of intra and extracranial stenotic diseases, and to correlate its occurrence with therapeutic responses.

The second phenomena we observed was the occurrence of Rankine vortex and its effect on cerebral blood flow. This vortex occurs in nature in Tornadoes^37^ and it is well studied in the field of combustion aerodynamics^38^. The circle of Willis is characterized by highly tortuous morphology, which dictates swirl and helical flow structure to the blood flow field^39^. When the flow passes through a region of high tortuosity, the streamlines (which are locally tangent to the velocity vector field) undergoes spatial curvature creating regions of forced vortices which is sometimes called *helical structures*^40^. In the case of pulsatile flow, the local acceleration term increases the swirl generation effects. When the swirl flow pattern is characterized by a forced vortex core and free vortex outer ring it is called Rankine vortex^41,42^. Rankine vortex is vitally important in many engineering^43^ and natural^44^ phenomena. In the medical literature, Rankine vortex was not reported in patient specific or idealized vascular geometries or in cerebrovascular hemodynamics to the best of the authors’ knowledge. Rankine vortex, however, was reported in few experimental PIV studies examining medical devices (heart valves)^45,46^. Rankine vortex is characterized by radial pressure gradient which compels the mass transfer through the vortex to pass tangent to its axis, and also by highly curved streamlines which increases the viscous dissipation towards its outer ring. The term “Rankine vortex” was not discussed in the medical literature previously, which opens the possibilities to study it in different scenarios and observe the contribution of complex vortex structures to different disease pathologies.

The third phenomenon was the observation of dynamic changes in viscosity influenced by the complex arterial geometry of the ICA in our MMD cohort. This dynamic viscosity changes led to areas of extreme increase in viscosity alternating with areas of lowered viscosity. These areas of increased viscosity can lead to blood stagnation, platelet aggregation and the development of emboli, thus leading to cerebral ischemia^47^. While the third phenomenon can be addressed using antiplatelet, the first two phenomena are pure hemodynamic phenomena that are physical in nature. Thus, antiplatelet therapy will indeed fail to tackle the hemodynamic disturbances caused by them and rerouting of the blood flow via bypass surgery will be the only valid option to correct the hemodynamic insufficiency. Moreover, how much of a difference medical therapy will make in cases where the flow insufficiency is due to high viscosity induced flow stagnation in early-stage MMD, or in intracranial stenosis generally, is still undetermined.

It is very important to understand why anti-platelets and/or anticoagulants are expected to fail in preventing PBH in particular. Aspirin, for example, has effects on both blood coagulability and rheology^48^. However, these two action lines are weakly connected. Rosenson *et al*.^49^ showed that the antithrombotic effects of Aspirin is not a result of changes in blood viscosity or rheological behavior. Additionally, the effect of anti-platelets on whole blood viscosity (WBV) is negligible at shear rate levels found in the present study. Shear rate levels in the present study, as spatiotemporal average over the five cases, are within 400 s-1 to 800 s-1 in the regions of interest. Corresponding to such variation in shear rate, the difference in WBV changes within 0.00175 Pa.s to 0.0055 Pa.s in average(0.5 ≤ ψ ≤ 1.5). On the other hand, in a prospective clinical trial, Lee *et al*.^50^ showed that Aspirin and Warfarin alter WBV by approximately 0.00042 Pa.s and 0.00024 Pa.s, for a shear-rate difference from 300 to 1000 s-1, respectively. Ma *et al*.^51^ found that Aspirin have noticeable effects on WBV at low shear rates (5 s-1) and negligible effects at medium and high shear rates (100 s-1, 200 s-1). Hence, we argue that the effect of anti-platelets and anticoagulants medication on blood viscosity is too small compared to the inertial-viscous interactions associated with PBH.

In this study we used the same inflow conditions and viscosity model for all the cases analyzed. However, how much will patient-specific flow waveforms and patient-specific viscosity measures would impact the degree of the phenomena observed is not yet determined. While the existence of the phenomena in their essence would not change, the degree by which each phenomenon affects the flow insufficiency may change in a quantitative manner when using patient specific data. This should be taken in consideration when reviewing the results of this work. The small number of patients is also another limitation, brought about by the rarity of MMD and especially early-stage MMD patients presenting by such progressive symptoms. Since CFD analysis would actually be pointless in advanced MMD cases when the ICA terminus is occluded totally, only the cohort of early-stage MMD patients can be analyzed using CFD. Given the equivocal nature of the diagnosis in many of these patients, especially prior to surgery, patient selection is indeed problematic, resulting in very few cases available for analysis. This also lead to the absence of a control group (asymptomatic early MMD) as these patients are almost never diagnosed until they become symptomatic. These limitations however do not negate the interesting and novel findings in this report, which can be applied to not only MMD, but any form of intra or extracranial stenosis can benefit from our presented CFD methodology and analysis.

In conclusion, our data shed an important light on the extreme complexities of blood hemodynamics in early-stage MMD and subsequently in intracranial stenosis at large. We show for the first time a reasoning for non-responsiveness to antiplatelet therapy that could be based on blood hemodynamics phenomena in this cohort of patients. Our data strongly suggest the importance of CFD in the clinical setting, and how it can be used to predict patient’s response to medical therapy in cases of conservative therapeutic decision in intracranial stenosis patients. Moreover, our simulation methodology and results are in-line with our previous findings and recommendations for the usage of non-Newtonian viscosity models in CFD simulations and clearly shows the value of abandoning the over-simplification brought about by adopting the Newtonian viscosity modeling currently used in most medical CFD simulations^12^. Indeed, we do not argue that hemodynamic studies of the CBF such as MR-SPECT should have a lesser importance, on the contrary, we employed these techniques as the gold standard for clinical diagnosis. Our aim was to take the CFD a step further towards its ultimate clinical goal as a clinical diagnostic and decision-making tool^12^. In this work we clearly demonstrate the power of CFD as decision making tool, even though the study was performed retrospectively, as CFD analysis was able to predict the root causes of hemodynamic insufficiency in our patient cohort, and to demonstrate why they did not respond to medical therapy. This value was only brought about by utilizing high-fidelity CFD, abandoning simplified modeling and searching for complex physical phenomena that were otherwise understudied or not reported. Future studies with more cases and in different disease scenarios should be able to discover more interesting phenomena that can help us understand the symptomatology, disease progression and to develop therapies directed towards these particular phenomena using novel strategies.

## Materials and Methods

### Collection of patient data and inclusion criteria

We attempted retrospective analysis of the Kohnan hospital database for patients with early-stage MMD (Suzuki’s angiographic staging of stage 1 and 2^1^) undergoing revascularization surgery^1^. Inclusion criteria were: 1) diagnosis of early-stage MMD by catheter angiography, 2) presence of three-dimensional (3D) rotational angiography, 3) Presence of pre- and post-operative magnetic resonance (MR) imaging and N-isopropyl-p-[^123^I] iodoamphetamine single-photon emission computed tomography (^123^I-IMP-SPECT) studies documenting quantitative cerebral blood flow. Sixty-one consecutive adult patients (sixty-seven hemispheres, aged 17–66 years, mean 42.8) with MMD who underwent STA-MCA anastomosis with indirect pial synangiosis by a single surgeon (M.F.) between July 2017 and October 2018 were assessed for eligibility. Diagnosis of MMD was based on the diagnostic criteria of the Research Committee on Spontaneous Occlusion of the Circle of Wills of the Ministry of Health, Labor and Welfare of Japan^52^. Surgical indication for MMD included all of the following items: the presence of ischemic symptoms (minor completed stroke and/or transient ischemic attack) and/or history of posterior hemorrhage, apparent hemodynamic compromise on ^123^I-IMP-SPECT, independent activities of daily living (modified Rankin Scale scores 0–2), and the absence of major cerebral infarction exceeding the vascular territory of one major branch of the MCA. Among 61 patients (67 hemispheres), 11 patients matched the inclusion criteria of the early-stage MMD on the 12 operated hemispheres, while 5 patients (5 hemispheres) lacked the 3D rotational angiography and/or serial ^123^I-IMP-SPECT with quantification. Consequently, 5 patients (5 hemispheres) were enrolled in this study. The side selected for analysis was based on the symptomatology and quality of STL models created for CFD.

This study was approved by the ethical review board of Kohnan hospital, Sendai, Japan. An informed consent was acquired from all patients regarding the collection of data.

### Segmentation and STL models generation

Conventional digital subtraction and 3D rotational angiography had been performed using standard transfemoral catheterization with a biplanar unit (Innova 3131, GE Healthcare Japan). Images had been obtained during a 6-second injection of contrast agent and a 200° rotation, with imaging at 30 frames per second for 5 seconds. The 150 projection images were reconstructed into a 3D data set of 512 × 512 × 512 isotropic voxels covering a field of view of 200 mm in all 3 directions^13,17^. DICOM files of the 3D angiography were then imported into Materialise Mimics software (Materialise, NV, Belgium) for visualization, stereolithography (STL) model creation and initial fine-tuning. The initial models were exported to 3-Matic software (Materialise, NV, Belgium) for further correction and surface tuning. The rate of volume change was suppressed to ≤ 5% during the smoothing process^17^. The final models were outputted as STL format files.

### CFD procedure

#### Large eddy simulation of pulsatile non-Newtonian blood flow

Large Eddy Simulation (LES) has been used to simulate the five cases in this paper. The advantage of using LES comes from its ability to resolve different flow regimes simultaneously without the need of employing regime-specific turbulence or transitional models^53–55^. In pulsatile blood flow simulations, LES has shown numerous advantages in capturing transitional and turbulent flow physics^56–58^. LES solves a filtered form of the Navier-Stokes equation such that a filter function *G*(*x*, *x*′) is used to differentiate between the scale which are directly resolved (DNS) and the scales which are modeled using a sub-grid scale model (SGS). Hence, any filtered flow variable  can be expressed as:1

In its essence, LES is a coarser form of DNS that has the advantages of resolving most of the energy bearing scales, however with less computational resources than DNS. LES involves subgrid modeling that means that the vortices which have length scales smaller than the filter length scale are modeled, not resolved. The filtered incompressible Navier-Stokes equations are:

2 $$\frac{\partial {\bar{u}}_{i}}{\partial {x}_{i}}=0$$

3 $$\frac{\partial {\bar{u}}_{i}}{\partial t}+\frac{\partial ({\bar{u}}_{i}{\bar{u}}_{j})}{\partial {x}_{j}}=\frac{\partial {\sigma }_{ij}}{\partial {x}_{j}}-\frac{\partial \bar{p}}{\partial {x}_{i}}-\frac{\partial {\tau }_{ij}}{\partial {x}_{j}}$$

where ${\sigma }_{ij}$ is the viscous stress tensor defined as:

4 $${\sigma }_{ij}\equiv [\mu (\frac{\partial {\bar{u}}_{i}}{\partial {x}_{j}}+\frac{\partial {\bar{u}}_{j}}{\partial {x}_{i}})]-\frac{2}{3}\mu \frac{\partial {\bar{u}}_{i}}{\partial {x}_{i}}{\delta }_{ij}$$

and ${\tau }_{ij}$ is the subgrid stress tensor defined as:

5 $${\tau }_{ij}\equiv \rho (\overline{{u}_{i}{u}_{j}}-{\bar{u}}_{i}{\bar{u}}_{j})$$

We have recently shown that the Newtonian assumption used in CFD models of cerebral blood flow is an inappropriate over-relaxed assumption^12,59^. To model the non-Newtonian shear thinning blood properties, the Carreau-Yasuda^60^ model was used. This model is widely used in cerebral blood flow simulations^61,62^. The model calculates the effective local instantaneous viscosity $\mu (x,t)$ as:

6 $$\mu (x,t)={\mu }_{\infty }+({\mu }_{0}-{\mu }_{\infty }){[1+{(\lambda \dot{\gamma })}^{a}]}^{\frac{n-1}{a}}$$

where *μ*_*∞*_ = 0.0022 Pa.s, *μ*_0_ = 0.022 Pa.s, *λ*=0.11 s, *a* = 0.644, n = 0.392 and $\dot{\gamma }$ is the local instantaneous shear rate magnitude. To provide a meaningful dimensionless measure of shear-thinning effects, we introduce $\psi (x,t)=\frac{\mu (x,t)}{{\mu }_{N}}$ where ${\mu }_{N}$ is the Newtonian viscosity $({\mu }_{N}=0.0035\,pa.s)$.

By definition, LES is mesh dependent. In such case, the application of LES mesh resolution quality measures is necessary to provide evidence of validity for the results. Pope^63^ proposed the measure of LES turbulence resolution $M(x,t)$ and showed that such measure provides valid ground for assessing the quality and validity of LES. Saqr^38,64^ and Saqr *et al*.^20,53,65,66^ developed a form of Pope’s LES resolution criterion that is suitable for application in any CFD code and tailored for the Smagorinsky-Lilly SGS model. This form has been utilized in the present work to ensure that LES resolved at least 80% of the total kinetic energy budget in all cases. It is expressed as:

7 $$M(x,t)=\frac{{K}_{res}(x,t)}{{K}_{res}(x,t)+{K}_{SGS}(x,t)}$$

where the resolved turbulence kinetic energy is calculated as:

8 $${K}_{res}(x,t)=\frac{1}{2}({\tilde{u}}_{i}^{2}+{\tilde{u}}_{j}^{2}+{\tilde{u}}_{k}^{2})$$

and the subgrid turbulence kinetic energy of the Smagorinsky-Lilly model is calculated as:

9 $${K}_{SGS}(x,t)=\frac{{L}_{s}^{2}|\bar{S}|}{{({C}_{s}\Delta )}^{2}}$$

where ${L}_{S}$ is the mixing length of the subgrid scales, calculated as ${L}_{S}=min[kd,{C}_{s}\Delta ]$ where $k$ is the *Von Karaman* constant, $d$ is the distance from the nearest cell wall, and ${C}_{s}\,$is the Smagorinsky constant and $|\bar{S}|=\sqrt{2{S}_{ij}{S}_{ij}}$ where ${S}_{ij}$ is the rate of strain tensor, and $\Delta$ is the grid filter length. Results of applying the LES quality measures given by Eqs. (6–8) are shown in Supplementary Fig. 1 where it is shown that in more than 90% of the grid cells $\,\overline{M(x,t)} > 0.9$.

#### Grid generation, boundary conditions and LES solver settings

The computational grids were created using ANSYS ICEM software with proper boundary-layer grid refinement, as shown in Fig. 1. The smallest grid cells were located in the near-wall region with first cell thickness of 5 *μm* to provide meaningful flow physics with scales corresponding to the size of endothelial cells. The number of grid cells ranged from $5.9\times {10}^{5}$ to $8.7\times {10}^{5}$ cells depending on the case. Since the flow is dominated by large structures and not a fully developed turbulent flow, the Kolmogorov scales are not persistently dominant. In other words, the flow in hand is not a homogenous isotropic turbulent flow, hence, the use of Taylor’s hypothesis and Kolmogorov scaling laws are not adequate. The supplementary materials contain the details of the computational grids and scale analysis of the flow (Supplementary Tables 1 and 2).

The boundary conditions used in the present work is of Womersley type with the following form:

10 $${u}_{c}(t)={U}_{o}+\mathop{\sum }\limits_{n=1}^{3}{A}_{n}\,\sin (n\omega t)+{B}_{n}\,\cos (n\omega t)$$

where ${u}_{o}(t)$ is the transient centerline velocity, ${U}_{o}$ is the steady component of the flow, ${A}_{n}$ and ${B}_{n}$ are the Fourier coefficients, $\omega$ is the angular velocity, and $t$ is time in seconds. The frequency was set to $f=1.16\,Hz$ and the total time simulated was set to 3 seconds. The coupled LES solver of ANSYS Fluent V16 was used to conduct the analysis. The solver utilizes central differencing scheme for discretizing the space derivatives and second order implicit time stepping for the local acceleration term. Further details of the numerical schemes and approaches used in this solver can be found in ANSYS Fluent Theory Guide.

## Supplementary information

Supplementary data.

## References

[1] Suzuki J, Takaku A (1969). Cerebrovascular “moyamoya” disease. Disease showing abnormal net-like vessels in base of brain. Archives of neurology.

[2] Kuriyama S (2008). Prevalence and clinicoepidemiological features of moyamoya disease in Japan: findings from a nationwide epidemiological survey. Stroke.

[3] Rashad S, Fujimura M, Niizuma K, Endo H, Tominaga T (2016). Long-term follow-up of pediatric moyamoya disease treated by combined direct-indirect revascularization surgery: single institute experience with surgical and perioperative management. Neurosurgical review.

[4] Fujimura M, Bang OY, Kim JS (2016). Moyamoya Disease. Front Neurol. Neurosci..

[5] Occlusion RCOTPATOSOOTCOWHLSRGFROMFIDTOS, Health Labour Sciences Research Grant for Research on Measures for Infractable, D (2012). Guidelines for diagnosis and treatment of moyamoya disease (spontaneous occlusion of the circle of Willis). Neurol. Med. Chir. (Tokyo).

[6] Fujimura M, Tominaga T (2012). Lessons learned from moyamoya disease: outcome of direct/indirect revascularization surgery for 150 affected hemispheres. Neurol Med Chir (Tokyo).

[7] Kaku Y (2012). Outer-diameter narrowing of the internal carotid and middle cerebral arteries in moyamoya disease detected on 3D constructive interference in steady-state MR image: is arterial constrictive remodeling a major pathogenesis?. Acta neurochirurgica.

[8] Ryoo S (2014). High-resolution magnetic resonance wall imaging findings of Moyamoya disease. Stroke.

[9] Yuan M (2015). High-resolution MR imaging of the arterial wall in moyamoya disease. Neuroscience letters.

[10] Oka K, Yamashita M, Sadoshima S, Tanaka K (1981). Cerebral haemorrhage in Moyamoya disease at autopsy. Virchows Arch A Pathol Anat Histol.

[11] Takagi Y, Kikuta K, Nozaki K, Hashimoto N (2007). Histological features of middle cerebral arteries from patients treated for Moyamoya disease. Neurol Med Chir (Tokyo).

[12] Saqr, K. M. *et al*. What does computational fluid dynamics tell us about intracranial aneurysms? A meta-analysis and critical review. *Journal of cerebral blood flow and metabolism: official journal of the International Society of Cerebral Blood Flow and Metabolism* **In-press**, 271678×19854640, 10.1177/0271678x19854640 (2019). 10.1177/0271678X19854640 PMC7181089 31213162

[13] Sugiyama S (2016). Blood Flow Into Basilar Tip Aneurysms: A Predictor for Recanalization After Coil Embolization. Stroke.

[14] Liu J (2017). Functional assessment of cerebral artery stenosis: A pilot study based on computational fluid dynamics. Journal of cerebral blood flow and metabolism: official journal of the International Society of Cerebral Blood Flow and Metabolism.

[15] Kamada, H., Imai, Y., Nakamura, M., Ishikawa, T. & Yamaguchi, T. Shear-induced platelet aggregation and distribution of thrombogenesis at stenotic vessels. *Microcirculation* **24**, 10.1111/micc.12355 (2017). 10.1111/micc.12355 28109051

[16] Marshall I, Zhao S, Papathanasopoulou P, Hoskins P, Xu Y (2004). MRI and CFD studies of pulsatile flow in healthy and stenosed carotid bifurcation models. Journal of biomechanics.

[17] Rashad S (2018). Impact of bifurcation angle and inflow coefficient on the rupture risk of bifurcation type basilar artery tip aneurysms. Journal of neurosurgery.

[18] Saqr KM, Wahid MA (2014). Effects of swirl intensity on heat transfer and entropy generation in turbulent decaying swirl flow. Appl. Therm. Eng..

[19] Saqr KM, Kassem HI, Aly HS, Wahid MA (2012). Computational study of decaying annular vortex flow using the R ε/k-ε turbulence model. Appl. Math. Model..

[20] Eldrainy YA, Saqr KM, Aly HS, Lazim TM, Jaafar MNM (2011). Large eddy simulation and preliminary modeling of the flow downstream a variable geometry swirler for gas turbine combustors. Int. Commun. Heat Mass Transf..

[21] Tu XK (2017). Uneven cerebral hemodynamic change as a cause of neurological deterioration in the acute stage after direct revascularization for moyamoya disease: cerebral hyperperfusion and remote ischemia caused by the ‘watershed shift’. Neurosurgical review.

[22] Fujimura M, Tominaga T (2015). Current status of revascularization surgery for moyamoya disease: special consideration for its ‘internal carotid-external carotid (IC-EC) conversion’ as the physiological reorganization system. Tohoku. J. Exp. Med..

[23] Kataoka H (2015). Results of Prospective Cohort Study on Symptomatic Cerebrovascular Occlusive Disease Showing Mild Hemodynamic Compromise [Japanese Extracranial-Intracranial Bypass Trial (JET)-2 Study]. Neurol. Med. Chir. (Tokyo).

[24] Mei CC, Jing H (2016). Pressure and wall shear stress in blood hammer–Analytical theory. Mathematical biosciences.

[25] Ghidaoui MS, Zhao M, McInnis DA, Axworthy DH (2005). A review of water hammer theory and practice. Applied Mechanics Reviews.

[26] Damşa T, Appel E, Cristidis V (1976). “Blood-hammer” phenomenon in cerebral hemodynamics. Mathematical Biosciences.

[27] Azhdari, M., Riasi, A. & Tazraei, P. Numerical Study of Non-Newtonian Effects on Fast Transient Flows in Helical Pipes. *arXiv preprint arXiv:1703.06877* (2017).

[28] Dubief Y, Delcayre F (2000). On coherent-vortex identification in turbulence. Journal of turbulence.

[29] Seybold HJ, Carmona HA, Herrmann HJ, Andrade JS (2019). Self-organization in purely viscous non-Newtonian turbulence. Physical Review Fluids.

[30] Gijsen F, Allanic E, Van de Vosse F, Janssen J (1999). The influence of the non-Newtonian properties of blood on the flow in large arteries: unsteady flow in a 90 curved tube. Journal of biomechanics.

[31] Liu X, Sun A, Fan Y, Deng X (2015). Physiological significance of helical flow in the arterial system and its potential clinical applications. Annals of biomedical engineering.

[32] Damşa, T., Appel, E. & Biosciences, C.-V. “Blood-hammer” phenomenon in cerebral hemodynamics. *Mathematical Biosciences* (1976).

[33] Chuiko GP, Dvornik OV, Shyian SI, Baganov YA (2018). Blood hammer phenomenon in human aorta: Theory and modeling. Mathematical Biosciences.

[34] Mei CC, Jing H (2018). Effects of thin plaque on blood hammer—An asymptotic theory. *European*. Journal of Mechanics, B/Fluids.

[35] Rossitti S (2015). The blood-hammer effect and aneurysmal basilar artery bifurcation angles. Journal of neurosurgery.

[36] Tazraei P, Riasi A, Takabi B (2015). The influence of the non-Newtonian properties of blood on blood-hammer through the posterior cerebral artery. Mathematical Biosciences.

[37] Knaff JA (2007). Statistical tropical cyclone wind radii prediction using climatology and persistence. Weather Forecast..

[38] Saqr, K. Turbulent Vortex Flames: Aerodynamics and Thermochemistry of Turbulent Confined Vortex Flames. (LAP LAMBERT Academic Publishing, 2011).

[39] Wetzel S (2007). *In vivo* assessment and visualization of intracranial arterial hemodynamics with flow-sensitized 4D MR imaging at 3T. Am J Neuroradiol.

[40] Vorobtsova N (2016). Effects of Vessel Tortuosity on Coronary Hemodynamics: An Idealized and Patient-Specific Computational Study. Annals of biomedical engineering.

[41] Ogawa, A. *Vortex Flow*. (Taylor & Francis, 1992).

[42] Ting, L., Klein, R. & Knio, O. M. *Vortex Dominated Flows: Analysis and Computation for Multiple Scale Phenomena*. (Springer Berlin Heidelberg, 2007).

[43] Saqr, K. M. *Aerodynamics and Thermochemistry of Turbulent Confined Asymmetric Vortex Flames*. (Universiti Teknologi Malaysia, 2011).

[44] Fiedler BH, Garfield GS (2010). Axisymmetric vortex simulations with various turbulence models. CFD Letters.

[45] Li CP, Chen SF, Lo CW, Lu PC (2012). Role of vortices in cavitation formation in the flow at the closure of a bileaflet mitral mechanical heart valve. J. Artif. Organs.

[46] Li CP, Lu PC, Liu JS, Lo CW, Hwang NH (2008). Role of vortices in cavitation formation in the flow across a mechanical heart valve. J Heart Valve Dis.

[47] Ranucci M (2015). Plasma viscosity, functional fibrinogen, and platelet reactivity in vascular surgery patients. Clin. Hemorheol. Microcirc..

[48] Elblbesy MA, Hereba ARM, Shawki MM (2012). Effects of aspirin on rheological properties of erythrocytes *in vitro*. Int. J. Biomed. Sci..

[49] Rosenson RS, Wolff D, Green D, Boss AH, Kensey KR (2004). Aspirin: Aspirin does not alter native blood viscosity. Journal of Thrombosis and Haemostasis.

[50] Lee C-H, Jung K-H, Cho DJ, Jeong S-K (2019). Effect of warfarin versus aspirin on blood viscosity in cardioembolic stroke with atrial fibrillation: a prospective clinical trial. BMC neurology.

[51] Ma N (2016). Evaluation on antithrombotic effect of aspirin eugenol ester from the view of platelet aggregation, hemorheology, TXB2/6-keto-PGF1α and blood biochemistry in rat model. BMC Veterinary Research.

[52] Tominaga T (2018). Recommendations for the Management of Moyamoya Disease: A Statement from Research Committee on Spontaneous Occlusion of the Circle of Willis (Moyamoya Disease) [2nd Edition]. Surgery for Cerebral Stroke.

[53] Aly HS, Saqr KM, Eldrainy YA, Jaafar MN (2009). Can large eddy simulation (LES) predict laminar to turbulent flow transition?. International Journal of Mechanical and Materials Engineering.

[54] Jiang J, Wang X (2012). Validation of Large Eddy Simulation in a relaminarizing boundary layer flow. CFD Letters.

[55] Sarris, I. E., Kassinos, S. C. & Carati, D. Large-eddy simulations of the turbulent Hartmann flow close to the transitional regime. *Physics of Fluids* **19**, 10.1063/1.2757710 (2007).

[56] Lantz J, Karlsson M (2012). Large eddy simulation of LDL surface concentration in a subject specific human aorta. Journal of biomechanics.

[57] Molla MM, Paul MC (2012). LES of non-Newtonian physiological blood flow in a model of arterial stenosis. Medical Engineering and Physics.

[58] Paul MC, Mamun Molla M, Roditi G (2009). Large-Eddy simulation of pulsatile blood flow. Medical Engineering and Physics.

[59] Saqr KM, Mansour O, Tupin S, Hassan T, Ohta M (2019). Evidence for non-Newtonian behavior of intracranial blood flow from Doppler ultrasonography measurements. Medical and Biological Engineering and Computing.

[60] Gijsen FJH, van de Vosse FN, Janssen JD (1999). The influence of the non-Newtonian properties of blood on the flow in large arteries: steady flow in a carotid bifurcation model. Journal of biomechanics.

[61] Bernabeu, M. O. *et al*. Impact of blood rheology on wall shear stress in a model of the middle cerebral artery. *Interface Focus* **3**, 10.1098/rsfs.2012.0094 (2013). 10.1098/rsfs.2012.0094 PMC3638489 24427534

[62] Bernsdorf J, Wang D (2009). Non-Newtonian blood flow simulation in cerebral aneurysms. Computers and Mathematics with Applications.

[63] Pope SB (2004). Ten questions concerning the large-eddy simulation of turbulent flows. New journal of Physics.

[64] Saqr KM (2010). Large Eddy Simulation: The demand for a universal measure of resolution. CFD Letters.

[65] Saqr, K. M., Aly, H. S., Kassem, H. I., Sies, M. M. & Wahid, M. A. In *International Conference on Theoretical and Applied Mechanics*, *International Conference on Fluid Mechanics and Heat and Mass Transfer - Proceedings*. 84–87.

[66] Saqr, K. M., Wahid, M. A. & Sies, M. M. In *AIP Conference Proceedings*. 400–408.

