# Author Correction: The hemodynamic complexities underlying transient ischemic attacks in early-stage Moyamoya disease: an exploratory CFD study

**DOI:** 10.1038/s41598-020-62862-7

**Published:** 2020-04-07

**Authors:** Sherif Rashad, Khalid M. Saqr, Miki Fujimura, Kuniyasu Niizuma, Teiji Tominaga

**Affiliations:** 1 Department of Neurosurgical Engineering and Translational Neuroscience, Tohoku University Graduate School of Medicine, Sendai, Miyagi 980-8575 Japan; 2 Department of Neurosurgery, Tohoku University Graduate School of Medicine, Sendai, Miyagi 980-8575 Japan; 3 Mechanical Engineering Department, College of Engineering and Technology, Arab Academy for Science, Technology and Maritime Transport, 1029 Abu-Kir, Alexandria Egypt; 4 Department of Neurosurgery, Kohnan hospital, Sendai, Miyagi Japan; 5 Department of Neurosurgical Engineering and Translational Neuroscience, Graduate School of Biomedical Engineering, Tohoku University, Sendai, Japan

Correction to: *Scientific Reports* 10.1038/s41598-020-60683-2, published online 28 February 2020

In the original version of this Article, Kuniyasu Niizuma was incorrectly affiliated with ‘Mechanical Engineering Department, College of Engineering and Technology, Arab Academy for Science, Technology and Maritime Transport, 1029, Abu-Kir, Alexandria, Egypt’.

The correct affiliations are listed below:

Affiliation 1:

Department of Neurosurgical Engineering and Translational Neuroscience, Tohoku University Graduate School of Medicine, Sendai, Miyagi, 980-8575, Japan

Affiliation 2:

Department of Neurosurgery, Tohoku University Graduate School of Medicine, Sendai, Miyagi, 980-8575, Japan

Affiliation 5:

Department of Neurosurgical Engineering and Translational Neuroscience, Graduate School of Biomedical Engineering, Tohoku University, Sendai, Japan

This error has now been corrected in the PDF and HTML versions of the Article. The accompanying Supplementary Information file was correct at the time of publication.

