# Supplementary material for: The hemodynamic complexities underlying transient ischemic attacks in early-stage Moyamoya disease: an exploratory CFD study
Source: Sci Rep. 2020 Feb 28;10:3700. doi: 10.1038/s41598-020-60683-2 (PMC7048746; doi:10.1038/s41598-020-60683-2)

# The hemodynamic complexities underlying transient ischemic attacks in early-stage Moyamoya disease: a CFD study

**Sherif Rashad, MD, PhD[1,2], Khalid M Saqr, PhD[3], Miki Fujimura, MD, PhD[4]. Kuniyasu Niizuma, MD, PhD[1,2,5]. Teiji Tominaga, MD, PhD[2].**

[1]Department of Neurosurgical Engineering and Translational Neuroscience, Tohoku University Graduate School of Medicine, Sendai, Miyagi, 980-8575, JAPAN.

[2]Department of Neurosurgery, Tohoku University Graduate School of Medicine, Sendai, Miyagi, 980-8575, JAPAN.

[3]Mechanical Engineering Department, College of Engineering and Technology, Arab Academy for Science, Technology and Maritime Transport, 1029 Abu-Kir, Alexandria, EGYPT

[4]Department of Neurosurgery, Kohnan hospital, Sendai, Miyagi, JAPAN.

[5]Department of Neurosurgical Engineering and Translational Neuroscience, Graduate School of Biomedical Engineering, Tohoku University, Sendai, JAPAN.

## Supplementary Materials

**Supplementary Table 1:** Main characteristic scales of the flow where mean Reynolds number ($Re_m$), Taylor microscale ($\lambda$), inner viscous scale ($\ell_{iv}$), Leipmann-Taylor scales ($\ell_{LT}$) and Kolmogorov scale ($\ell_K$) as calculated based on the boundary condition and the diameters of largest and smallest arteries in the five cases.

| **Case** | $d_{max}(10^{-3}m)$ | $d_{min}(10^{-3}m)$ | $Re_m$ | $\lambda\ (10^{-3}m)$ | $\ell_{iv}\ (10^{-3}m)$ | $\ell_{LT}(10^{-3}m)$ | $\ell_K(10^{-6}m)$ |
|---|---|---|---|---|---|---|---|
| 1 | 3.88 | 0.34 | 223.66 | 0.162 | 0.294 | 1.30 | 5.88 |
| 2 | 4.54 | 0.78 | 281.96 | 0.264 | 0.567 | 1.35 | 11.3 |
| 3 | 3.26 | 1.02 | 226.84 | 0.315 | 0.873 | 1.08 | 17.5 |
| 4 | 4.0 | 0.80 | 254.4 | 0.271 | 0.628 | 1.25 | 12.6 |
| 5 | 3.54 | 0.76 | 227.9 | 0.266 | 0.648 | 1.17 | 13.0 |

**Supplementary Table 2:** Details of computational grids where Δ is the LES filter length

| **Case** | **No. of grid cells** | $\Delta\ (10^{-6}m)$ | **Maximum orthogonal skewness** |
|---|---|---|---|
| Case 1 | 788,661 | 11.02 | 0.78 |
| Case 2 | 869,001 | 9.78 | 0.68 |
| Case 3 | 691,472 | 13.38 | 0.80 |
| Case 4 | 597,177 | 18.07 | 0.79 |
| Case 5 | 686,517 | 19.10 | 0.81 |

**Supplementary table 3:** Clinical details of the patients included in the study

| Case No. | Age | Sex | Side | Suzuki Grade | Presentation |
|---|---|---|---|---|---|
| 1 | 45 | M | R*<br>L | 2<br>0 | Ischemic infarction |
| 2 | 63 | F | R*<br>L | 2<br>3 | TIA not responsive to medical therapy |
| 3 | 36 | M | R*<br>L | 1<br>2 | Ischemic infarction |
| 4 | 47 | F | R<br>L* | 2<br>2 | TIA not responsive to medical therapy |
| 5 | 52 | M | R<br>L* | 0<br>2 | TIA not responsive to medical therapy |

**Asterisk:** Side analyzed in this study.

**Supplementary Figure 1:** Histogram of $\overline{M(x,t)}$ in the five CFD models showing the prevalence of the resolved scales in all cases where at least 80% of the cells has $\overline{M(x,t)} \geq 0.9$.

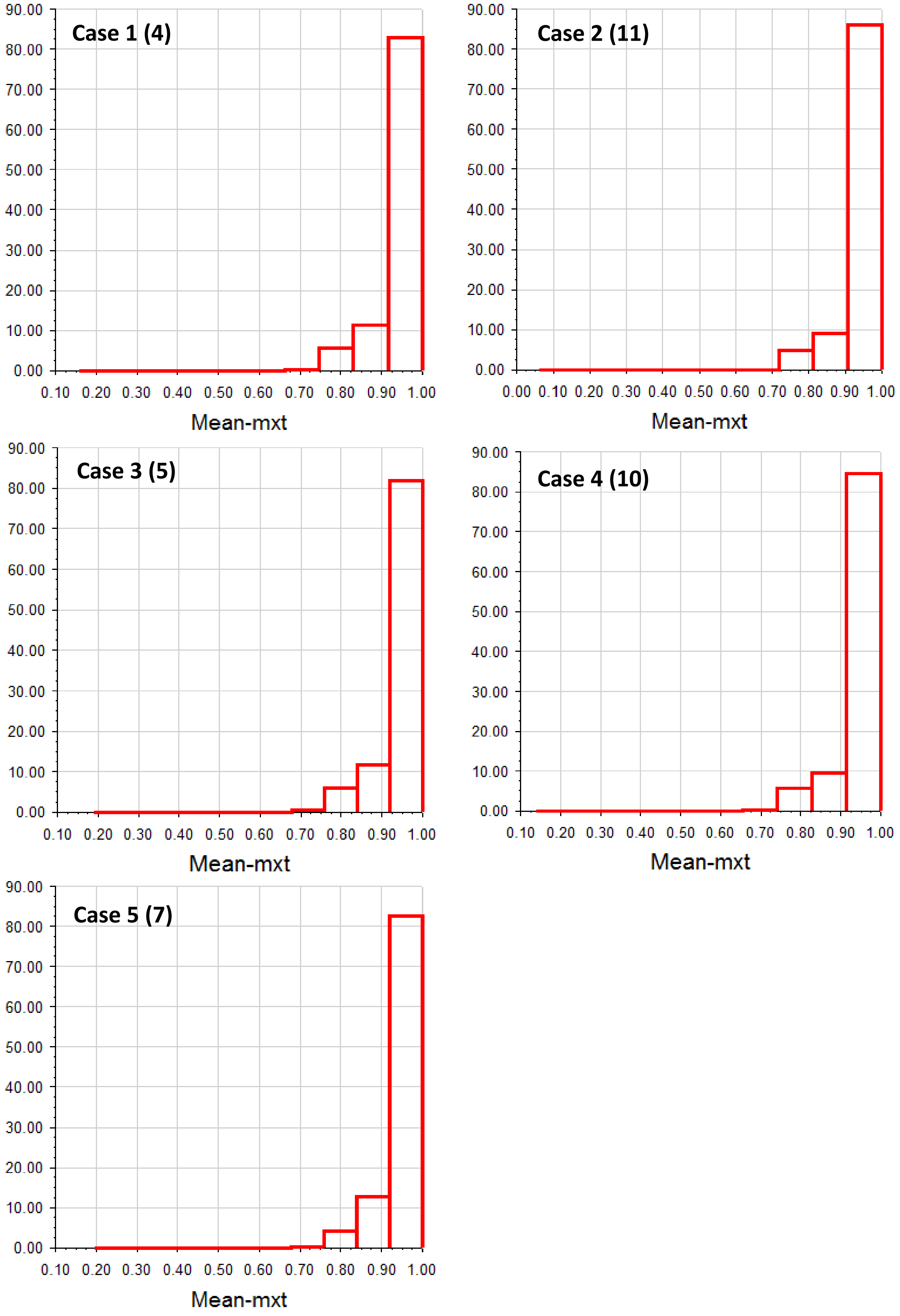

**Supplementary figure 2.** Case 1 clinical imaging. a) 2D diagnostic angiography showing right terminal ICA and MCA stenosis. No apparent MMD collateral vessels are observable at this stage. b) T2 MRI showing a small right ischemic infarction in the right MCA territory. c) MRI SPECT showing significant reduction in cerebral blood flow to the right cerebral hemisphere at rest. d) one year follow up MRA showing the still patent STA-MCA bypass and the progression of ICA stenosis with almost near complete MCA disappearance.

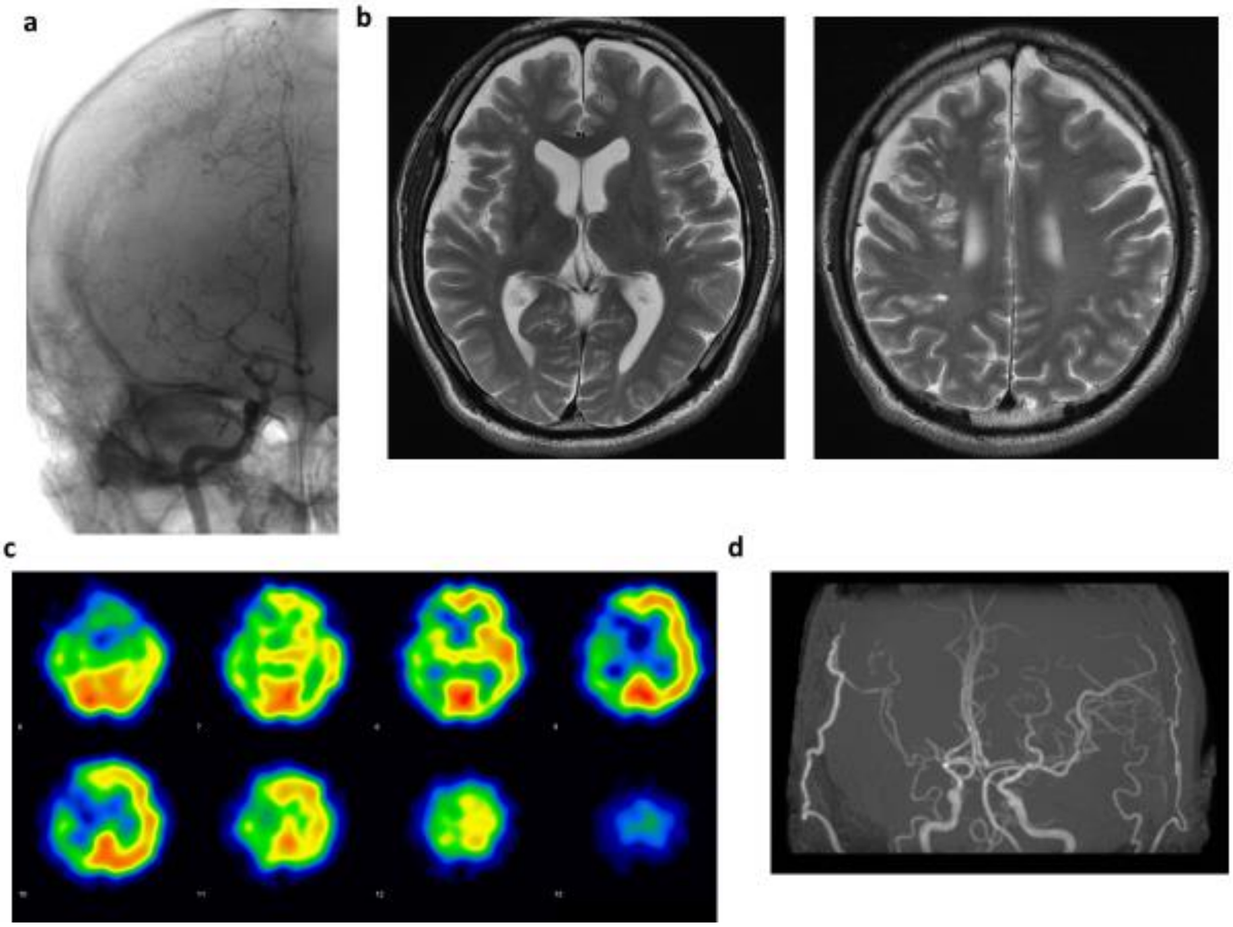

**Supplementary figure 3.** Screenshot from 3-matic software from different viewing angles showing the STL model created for Case 1 [Right Side]

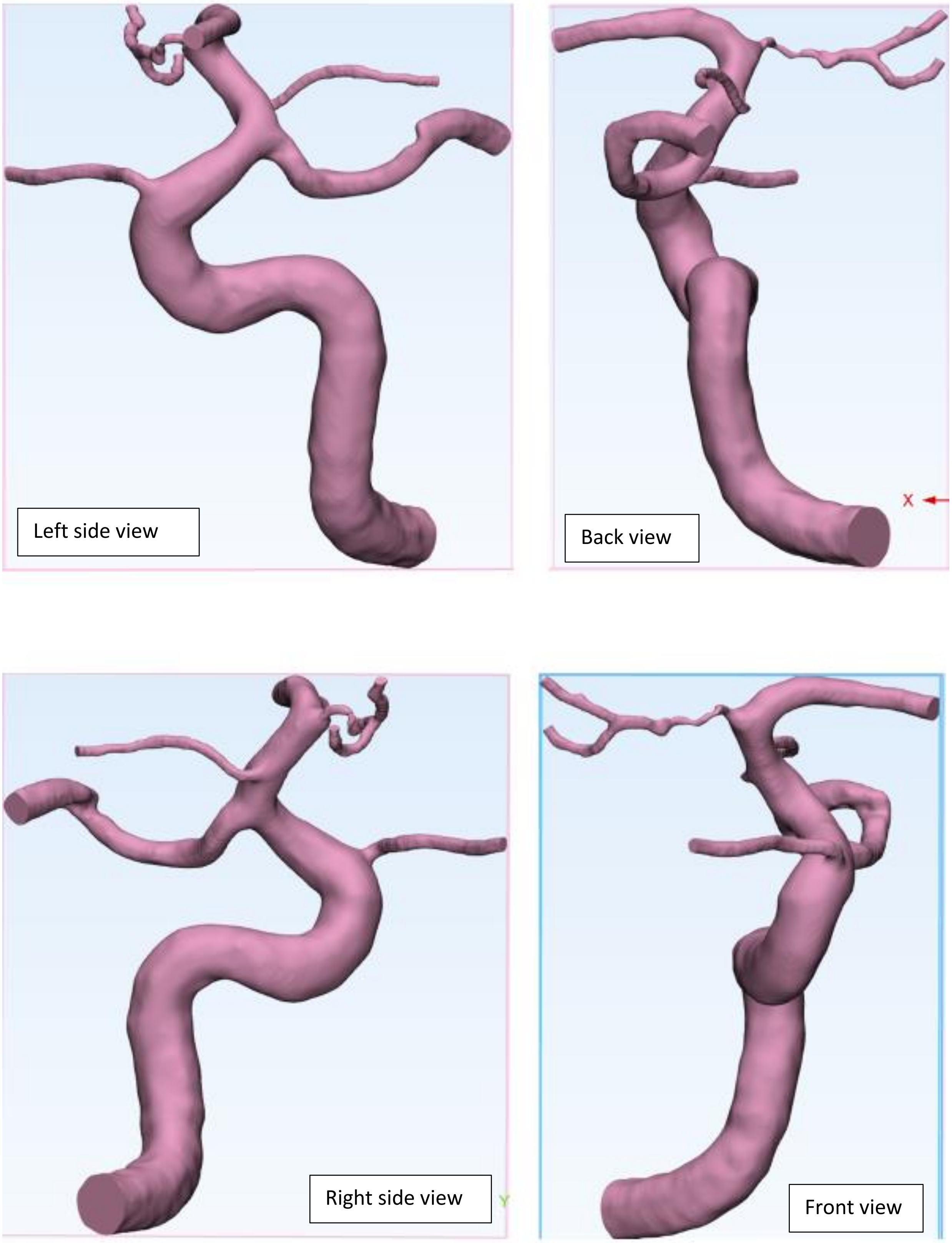

**Supplementary figure 4.** Case 2 clinical imaging. a) 2D diagnostic angiography showing the significant right MCA stenosis with the absence of the characteristic MMD puff of smoke collateral appearance. b) T2 MRI showing white matter affection on the right hemisphere. Notably this patient suffered from repeated TIA attacks. c) MRI SPECT showing the significant reduced blood flow to the right cerebral hemisphere at rest. d) one year follow up MRA showing the still patent STA-MCA bypass. The patient did not suffer any more TIA attacks following the surgery.

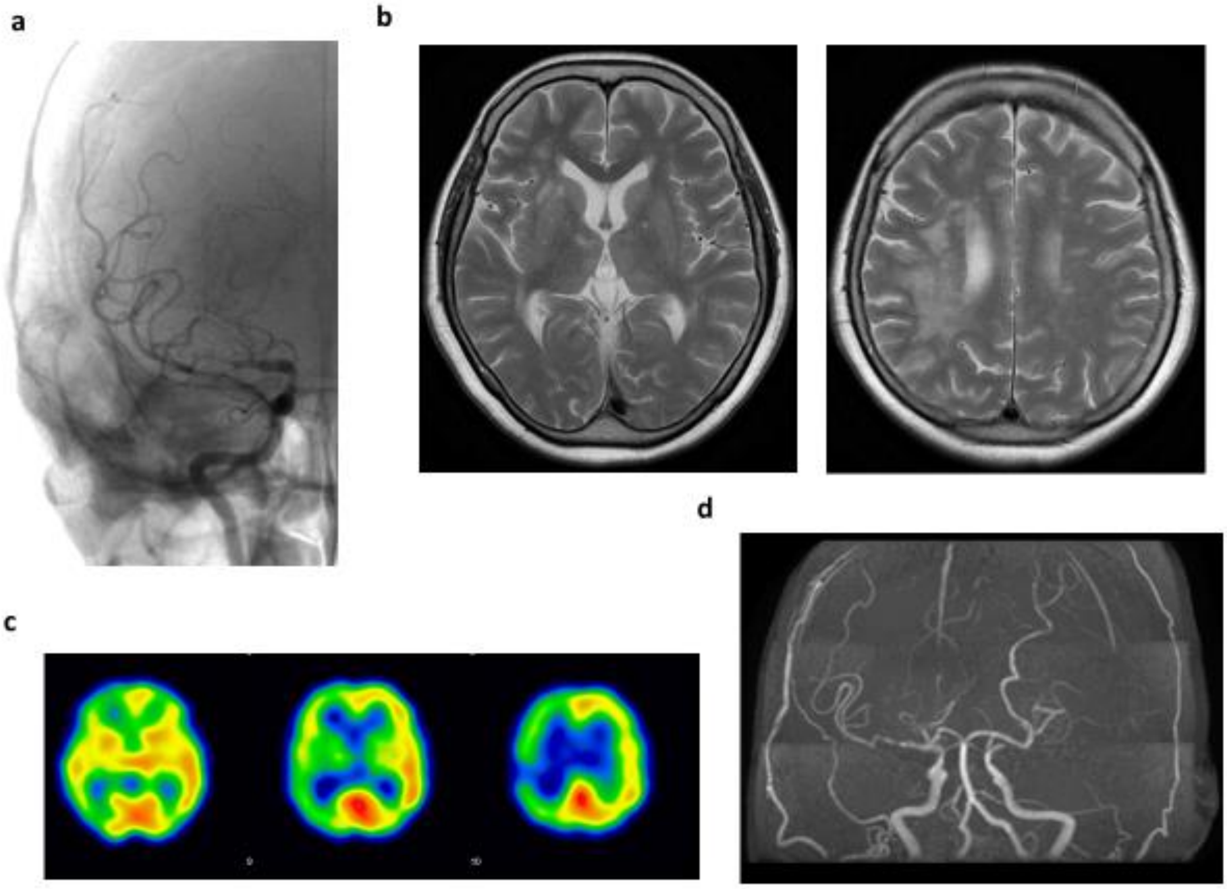

**Supplementary figure 5.** Screenshot from 3-matic software from different viewing angles showing the STL model created for Case 2 [Right side]

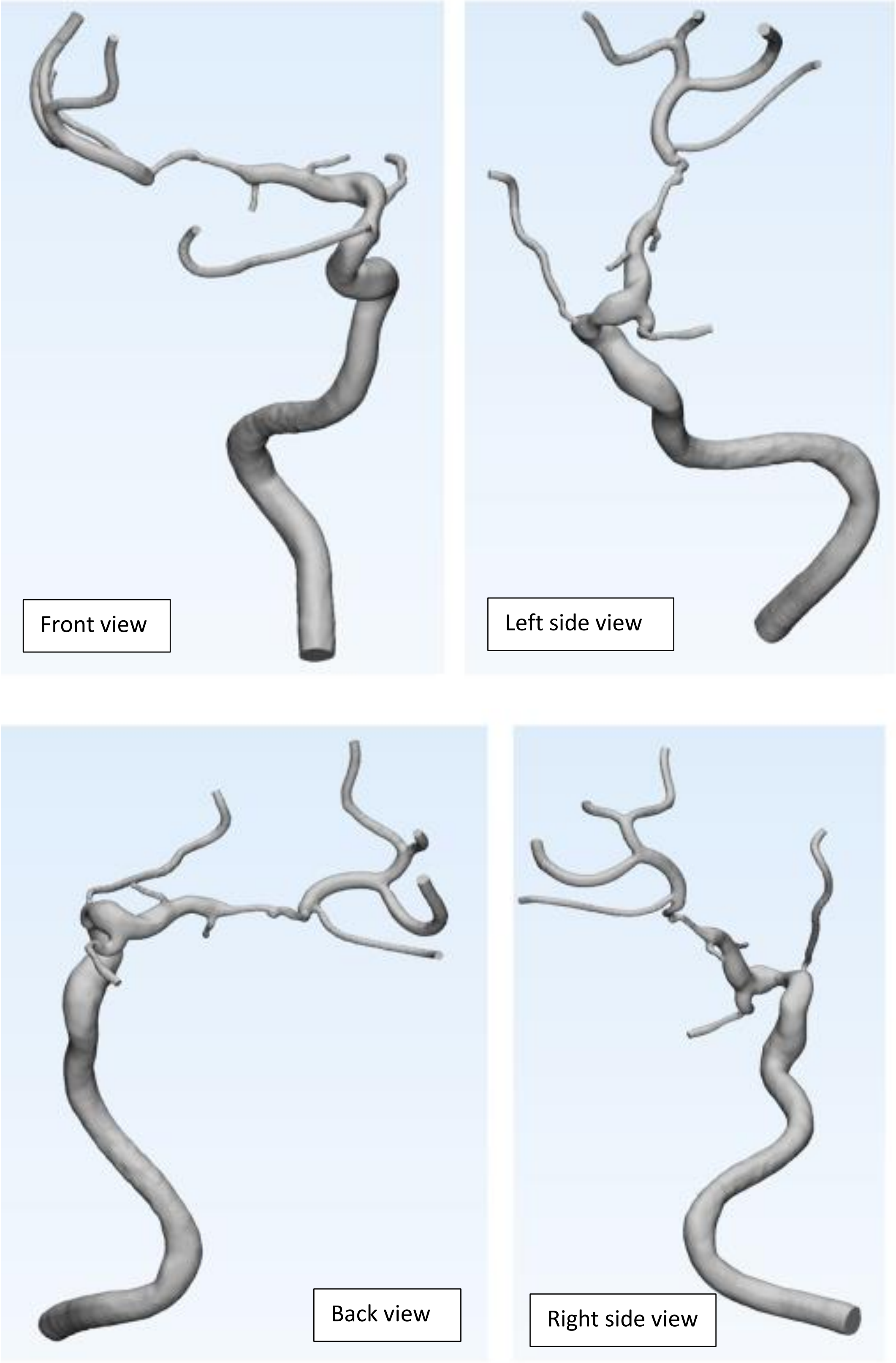

**Supplementary figure 6.** Case 3 clinical imaging. a) 2D diagnostic angiography showing the involvement of the right terminal ICA with significant narrowing of both ACA and MCA. This case also did not exhibit the MMD characteristic collaterals at the base of the brain. b) T2 MRI showing significant ischemic infarct affecting the right hemisphere in the territory of the MCA artery. c) MRI SPECT showing significant cerebral blood flow reduction to the right hemisphere. d) One year follow up MRA showing the still patent bypass and near occlusion of the terminal ICA on both sides. This patient had bilateral disease and the left STA-MCA bypass can also be viewed in this MRA.

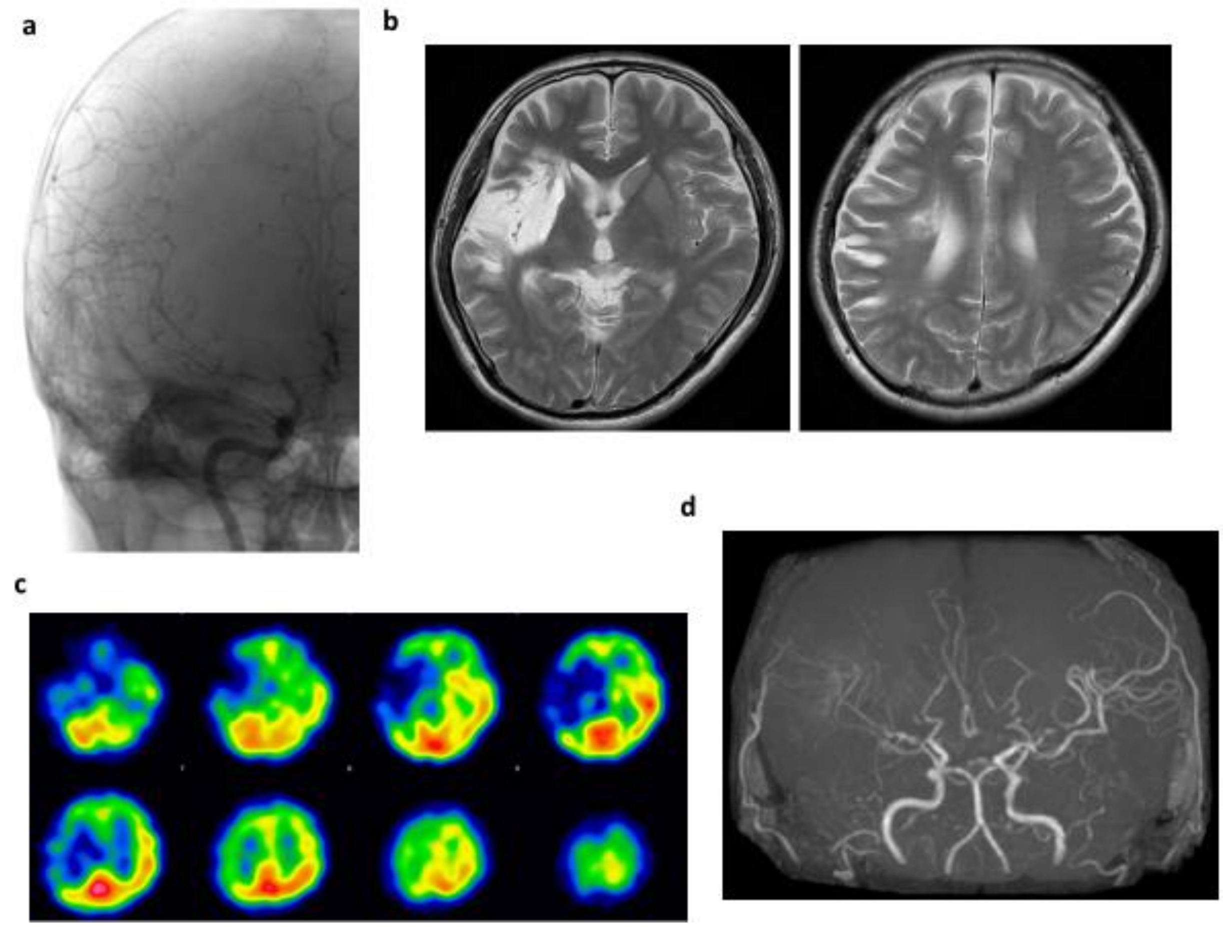

**Supplementary figure 7.** Screenshot from 3-matic software from different viewing angles showing the STL model created for Case 3 [Right side]

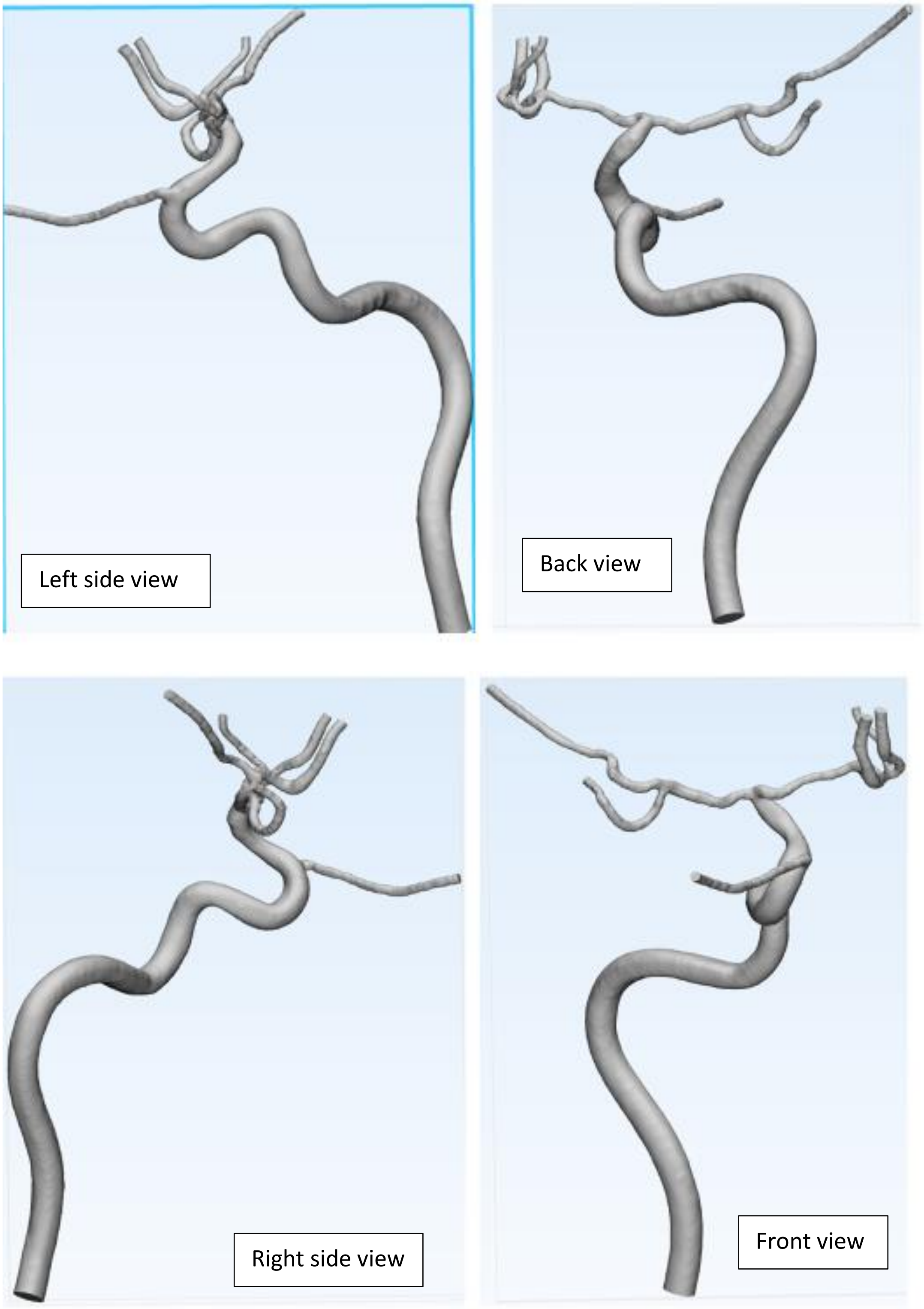

**Supplementary figure 8.** Case 4 clinical imaging. a) 2D diagnostic angiography showing the stenosis of the left terminal ICA and the involvement of ACA and MCA as well. No Moyamoya vessels are observable at the base of the brain. b) MRI showing remnant of small left white matter infarct. The patient suffered from repeated TIAs. c) MRI SPECT showing significant reduction of cerebral blood flow to the left cerebral hemisphere. d) One year follow up MRA showing the still patent bypass and near occlusion of the terminal ICA on both sides. This patient had bilateral disease and the right STA-MCA bypass can also be viewed.

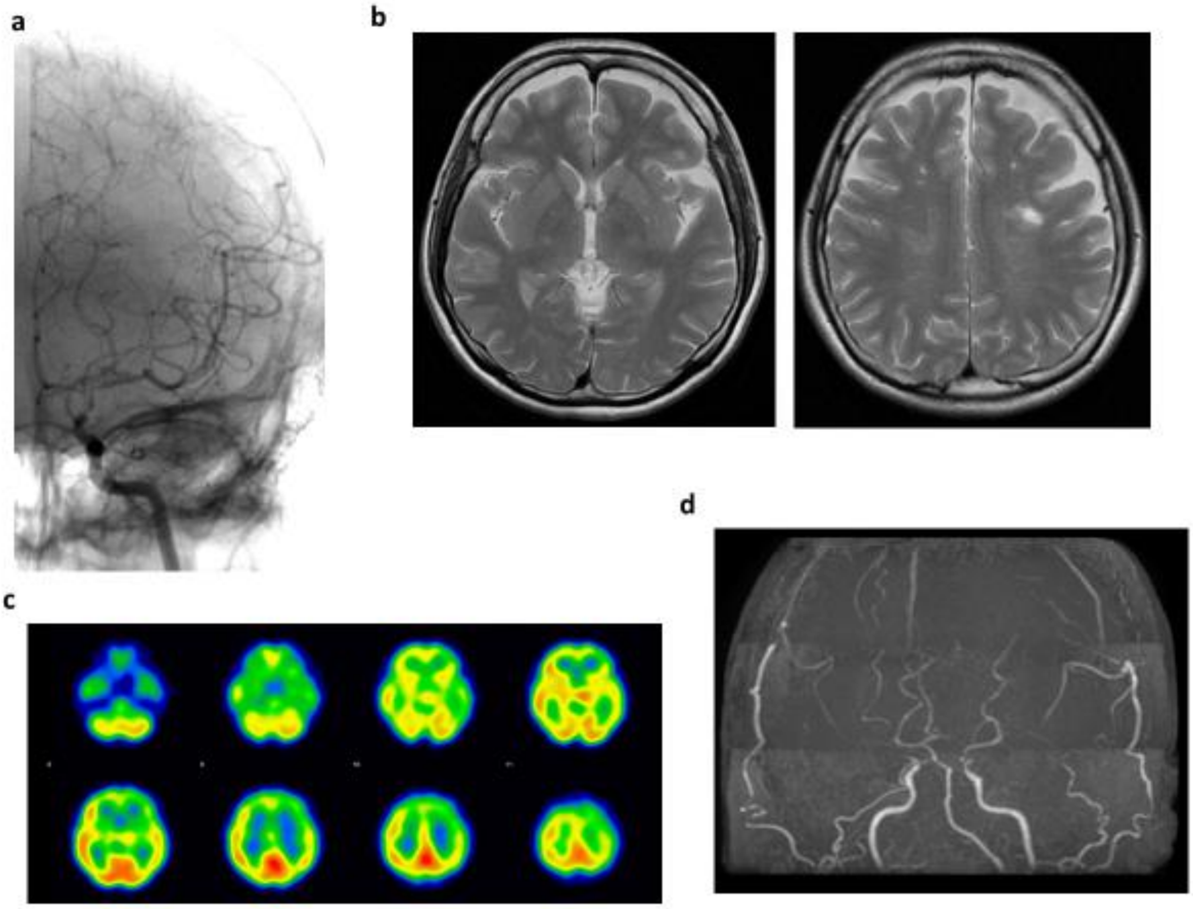

**Supplementary figure 9.** Screenshot from 3-matic software from different viewing angles showing the STL model created for Case 4 [Left side]

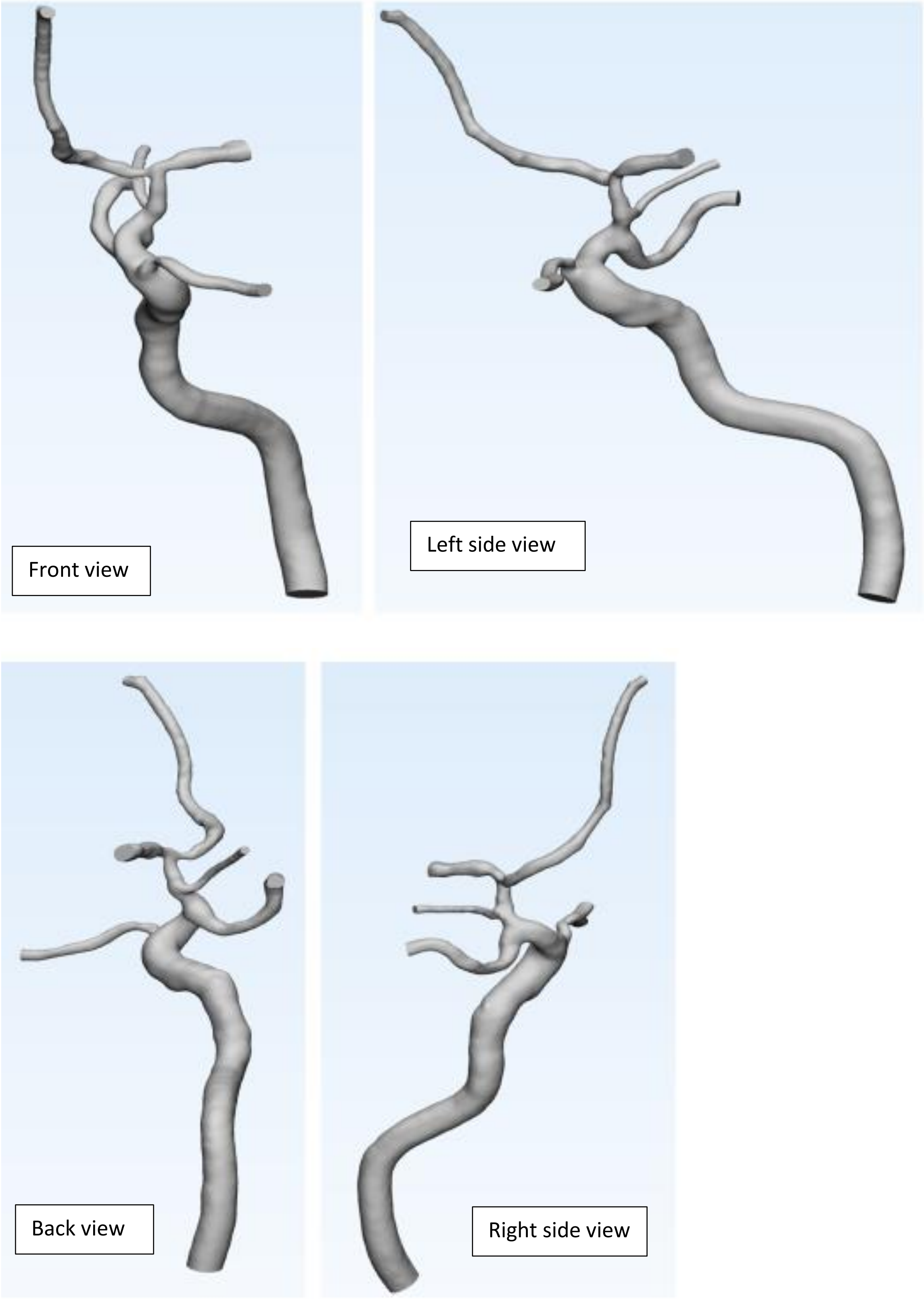

**Supplementary figure 10.** Case 5 clinical imaging. a) 2D diagnostic angiography showing the stenosis of the left terminal ICA. No Moyamoya vessels are observable at the base of the brain. b) MRI showing left white matter ischemia from repeated TIAs. c) MRI SPECT showing significant reduction of cerebral blood flow to the left cerebral hemisphere. d) One year follow up MRA showing the still patent bypass and complete occlusion of the terminal ICA on the left side.

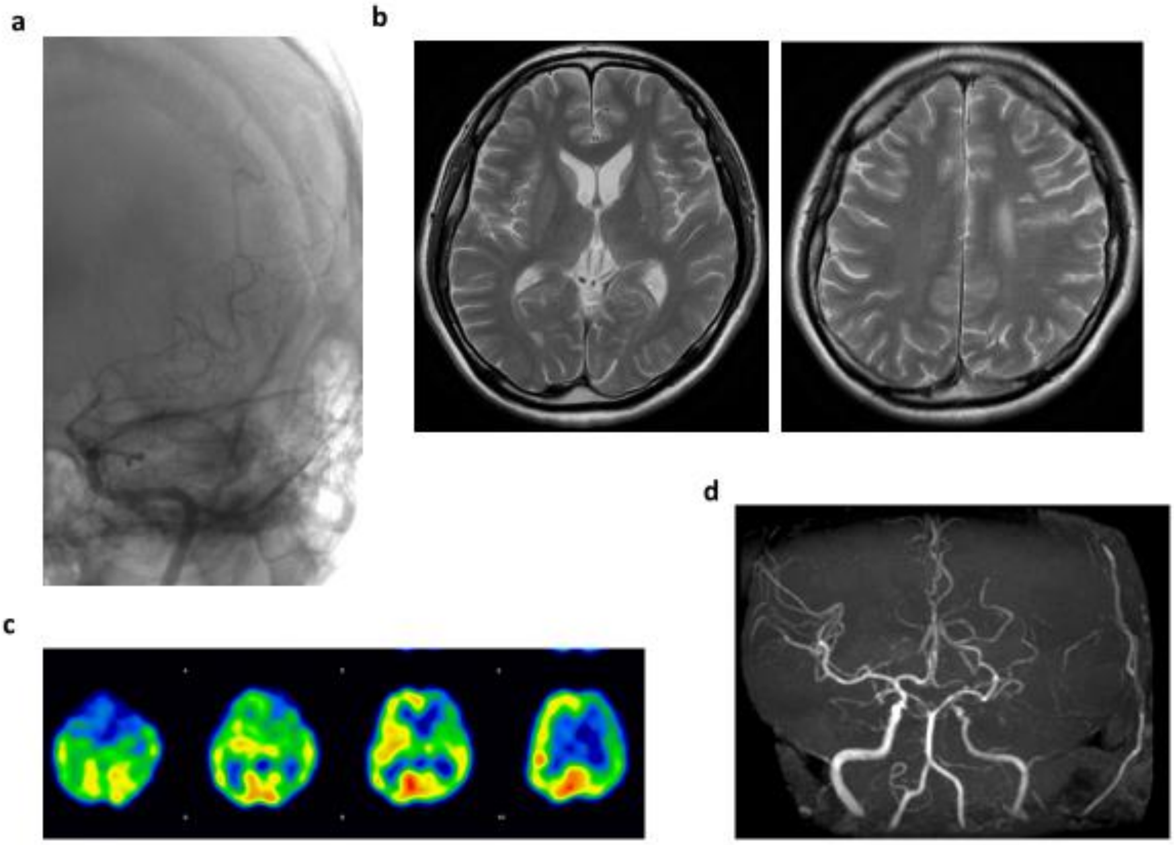

**Supplementary figure 11.** Screenshot from 3-matic software from different viewing angles showing the STL model created for Case 5 [Left side]

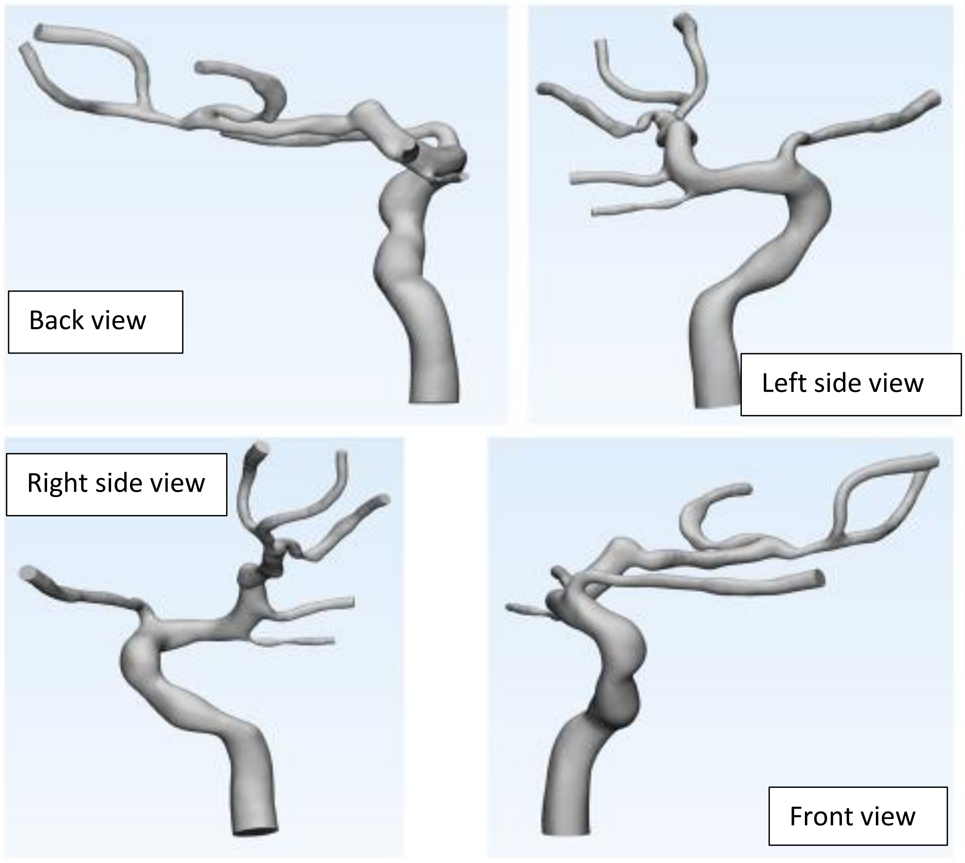

**Supplementary figure 12.** Case 1: Pressure loss coefficient $L = \frac{p_u(t)-p_d(t)}{\overline{p_u(t)}}$, where $p_u(t)$, $p_d(t)$ and $\overline{p_u(t)}$ is the time series of upstream, downstream pressure, and time-averaged upstream pressure, respectively. The $PL$ profile shows PBH at location P2 in comparison with non-PBH at location P3. In the figure, $PL(2) = \frac{P4(t)-p2(t)}{\overline{p4(t)}}$ is negative showing the transient pressure rise due to PBH at location P2. The phase-lag difference between the two time series approximately equals $\pi$ marking the drastic impact of PBH on the flow.

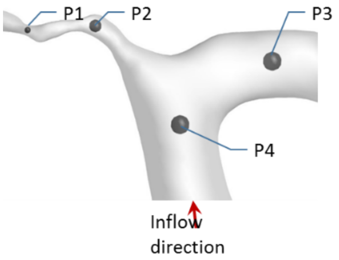


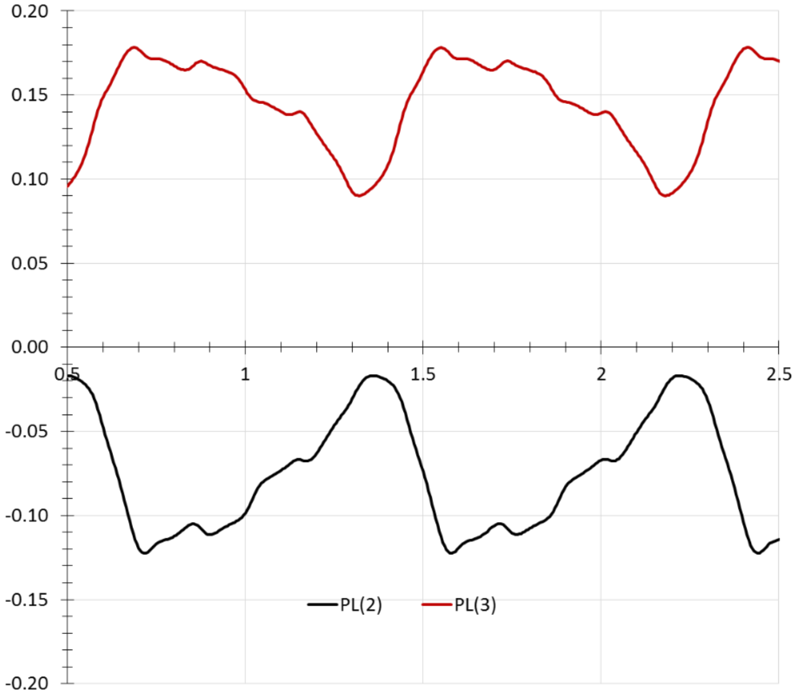

**Supplementary figure 13. Case 5: Flow separation at the MCA-AT bifurcation reduces blood flow to both arteries and creates persisting flow stagnation regions.** (a) time-averaged flow pathlines (b) mid-plane time-averaged velocity contours and vectors to highlight the persisting flow separation and stagnation zones at the MCA-AT bifurcation (c) mid-plane time-averaged velocity controus at the ACA showing flow separation and stagnation regions (d) distribution of blood flow rate at different branches

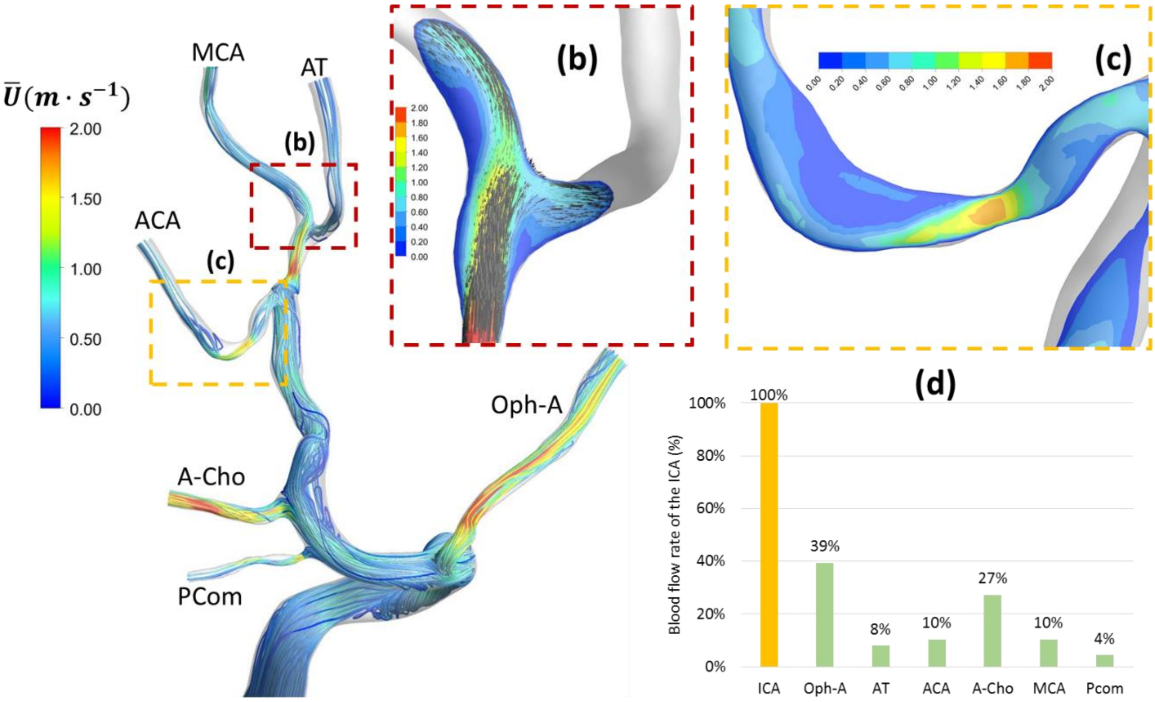

Supplement: Supplementary file 1 — Supplementary data. [file 41598_2020_60683_MOESM1_ESM.pdf]
